# UV-Cured Antibacterial Hydrogels Based on PEG and Monodisperse Heterofunctional Bis-MPA Dendrimers

**DOI:** 10.3390/molecules26082364

**Published:** 2021-04-19

**Authors:** Patrik Stenström, Yanmiao Fan, Yuning Zhang, Daniel Hutchinson, Sandra García-Gallego, Michael Malkoch

**Affiliations:** 1 Department of Fibre and Polymer Technology, Division of Coating Technology, KTH Royal Institute of Technology, Teknikringen 56-58, 100 44 Stockholm, Sweden; pstens@kth.se (P.S.); yanmiao@kth.se (Y.F.); yunzha@kth.se (Y.Z.); danhut@kth.se (D.H.); sandra.garciagallego@uah.es (S.G.-G.); 2 Department of Organic and Inorganic Chemistry, University of Alcalá, 28871 Madrid, Spain

**Keywords:** dendrimers, hydrogels, antibacterial, poly (ethylene glycol), thiol-ene

## Abstract

Bacterial infections are one of the major threats to human health due to the raising crisis of antibiotic resistance. Herein, second generation antibacterial heterofunctional dendrimers based on 2,2-bis(methylol)propionic acid were synthesized. The dendrimers possessed six alkenes and 12 ammonium end-groups per molecule and were used to fabricate antibacterial hydrogels together with dithiol-functional polyethylene glycol (mol wt of 2, 6 and 10 kDa) as crosslinkers via thiol-ene chemistry. The network formation can be completed within 10 s upon UV-irradiation as determined by the stabilization of the storage modulus in a rheometer. The hydrogels swelled in aqueous media and could be functionalized with the N-hydroxysuccinimide ester of the dye disperse red 13, which allowed for visually studying the degradation of the hydrogels through the hydrolysis of the ester bonds of the dendritic component. The maximum swelling ratio of the gels was recorded within 4–8 h and the swelling ratios increased with higher molecular weight of the polyethylene glycol crosslinker. The gel formed with 10 kDa polyethylene glycol crosslinker showed the highest swelling ratio of 40 and good mechanical properties, with a storage modulus of 8 kPa. In addition, the hydrogels exhibited good biocompatibility towards both human fibroblasts and mouse monocytes, while showing strong antibacterial activity against both gram-positive and gram-negative bacteria.

## 1. Introduction

Hydrogels are physically or chemically crosslinked networks that absorb water without dissolution due to their crosslinks, which prevent solubilization of their components. Their unique physiochemical properties have led to them having a wide range of applications in the field of biomedicine [1]. Hydrogel-based contact lenses are perhaps the most successfully commercialized example. These soft contact lenses, introduced by Czech chemist Otto Wichterle in the early 1960s [2], are considered by most users to be significantly more comfortable than harder lenses based on e.g., poly(methyl methacrylate) (PMMA). Another example of commercialized hydrogels is the superabsorbent polymers used in hygiene products such as disposable diapers [3].

By forming hydrogels with polymers containing functional groups that do not take part in the crosslinking reaction, the applications of these materials can be expanded even further. Such active and functional gels have been applied in the creation of self-healing hydrogels [3,4,5], antimicrobial wound dressings [6,7,8,9,10], hydrogels for drug-delivery [11,12,13] and in tissue engineering [14,15,16,17].

Dendrimers exhibit a large number of end-groups in relation to their size, which makes them excellent at interacting with their environment. Their monodispersity is advantageous in research since it enables unquestionable batch-to-batch consistency and the possibility to draw conclusions from results due to even small structural changes since the exact architecture is always known. Polyester dendrimers based on 2,2-bis(methylol)propionic acid (bis-MPA) have been available since the early 1990s [18] and have been shown to be biodegradable and biocompatible [19]. As of late, newer synthesis methods have emerged, enabling facile synthesis of bis-MPA dendrimers in decagram scales [20]. With the previous need for column chromatography steps now eliminated, these materials are far more accessible than they are perhaps reputed to be.

Dendrimers containing cationic end groups have been shown to exhibit antibacterial properties [20,21,22]. These are exciting materials as they are able to efficiently kill bacteria without employing antibiotics; therefore, their use can probably prevent the evolution of antibiotic resistant bacteria. Microbial infections caused by drug-resistant bacteria are a serious threat to human health due to their high mortality rate and high economic costs [21]. The prevalence of resistant bacteria has increased due to the overuse and misuse of commonly used antibiotics combined with a slowing rate of discovery of new antibiotics, due to a lack of investment by the pharmaceutical industry [22]. As a result, there is a crucial need for the development of novel antibacterial materials and new techniques to treat existing drug resistant bacteria, while also limiting further drug resistance.

In a recent paper, we presented the facile synthesis of a library of cationic amino/ammonium-functionalized bis-MPA dendrimers [23]. It was concluded that the second-generation dendrimer, possessing 12 ammonium-trifluoroacetate end-groups, exhibited good antibacterial properties while showing better biocompatibility than the higher generation dendrimers. In this article, this second-generation dendrimer is further developed by incorporating alkene groups into its constituent bis-MPA-derived monomers, resulting in monodisperse, second generation heterofunctional dendrimers with six alkene groups and 12 ammonium trifluoroacetate end-groups. Thiol functional polyethylene glycol (PEG) of varying mol wt were also synthesized and used as crosslinkers to form cationic ammonium functional hydrogels through UV initiated thiol-ene reactions with the alkene groups of the dendrimers. 

Thiol-ene chemistry has attracted increased attention in scientific research in recent years and the reaction is often considered to be a “click” reaction due to its high reaction rate and absolute atom economy [24]. The mechanism of the reaction is based on the formation of a thionyl radical, which can be obtained through a radical producing initiator. UV-active initiators are popular due to their capability of producing radicals under mild conditions such as ambient temperature and pressure. In this work, lithium phenyl-2,4,6-trimethylbenzoylphosphinate (LAP) was used as the photo-initiator since it is water soluble, biocompatible and readily produces radicals when irradiated at 365 nm [25].

Good biocompatibility is the most basic requirement for materials intended for use in medical applications and minimal damage should occur when the materials are in direct contact with human tissues. The biocompatibility and antimicrobial properties of the ammonium functional hydrogels were examined and compared to hydrogels formed using analogous hydroxyl functional dendrimers. The possibility of post-functionalizing the gels through swelling them with solutions of N-hydroxysuccinimide (NHS) esters was investigated. Relevant physical and chemical properties such as the gel fraction, swelling and rheological properties were determined.

The ammonium-functional dendrimers that inspired these formulations were shown to exhibit biodegradation through hydrolysis of their beta-alanine end-groups. In this work, experiments were conducted to verify whether this is true not only for the multifunctional dendrimers carrying both ammonium and allyl end-groups using matrix assisted laser desorption/ionization mass spectrometry (MALDI-MS), but also qualitatively for when these dendrimers are incorporated into gels through a staining method.

## 2. Materials and Methods

### 2.1. Chemicals

Bis-MPA was obtained in kind from Perstorp AB. The first-generation hydroxyl functional dendrimer was synthesized according to a previously published procedure [20]. PEG2k, PEG6k, PEG10k and mPEG5k were purchased from Sigma Aldrich (Sigma-Aldrich AB, Stockholm, Sweden). Carbonyldiimidazole (CDI) was purchased from Carbosynth (Carbosynth Ltd, United Kingdom). Cesium fluoride was purchased from Sigma Aldrich. 4-Pentenoic acid was purchased from Sigma Aldrich. Boc-protected beta-alanine was prepared according to previously published procedures [23,26]. Deuterated solvents were purchased from Cambridge Isotope Laboratories (Cambridge Isotope Laboratories, Tewksbury, MA, USA). Ethyl acetate, heptane, methanol, ethanol, palladium on carbon, butanol, benzyl alcohol, DHB and DCTB for MALDI were purchased from Sigma Aldrich. Deionized water was produced from tap water by reverse osmosis in a Eurowater Silhorko purification system, followed by deionization on a mixed ion exchange bed and purification by UV-lamps. The conductivity was 7 µS/cm. Dulbecco’s Modified Eagle Medium (DMEM), PBS, penicillin/streptomycin and tripsin-EDTA were purchased from Gibco. Human Dermal Fibroblasts (HDF) and mouse monocytes (RAW 264.7) were purchased from ATCC (American Tissue Culture Collection). The cell lines were maintained in DMEM containing 10% fetal bovine serum (FBS), penicillin G (100 IU) and streptomycin 100 mg/L under 5% CO_2_ at 37 °C. SalvequickMED Antibact Maxi Cover was purchased from Apotea AB, Sweden. 

### 2.2. Instruments and Methods

#### 2.2.1. NMR Spectroscopy

^1^H and ^13^C NMR spectroscopy was performed on a 400 MHz Bruker Avance III with a Bruker 5 mm BBFO SmartProbe^TM^ (Bruker Daltonics, Bremen, Germany). ^1^H NMR spectroscopy was performed using 32 scans, 1 s relaxation delay and 20 ppm spectral window. The respective values for ^13^C NMR spectroscopy were 512 scans, 2 s and 240 ppm. Spectra were collected in deuterated chloroform or deuterated methanol and were referenced to the internal solvent signal, with chemical shifts reported in δ units (ppm). The samples were analyzed in high throughout NMR tubes with a diameter of 5 mm from Wilmad-LabGlass. The sample volume was 0.7 mL. The spectra were analyzed with MestreNova v. 9.0.0-12821 from Mestrelab Research.

#### 2.2.2. MALDI-MS

MALDI-MS was carried out using a Bruker Ultraflex-III with a SCOUT-MTP Ion Source (Bruker Daltonics, Bremen, Germany). The instrument was calibrated using dendritic calibrants purchased from Polymer Factory AB, Stockholm, Sweden. Samples were prepared using a mass ratio of 1:1:40 of sample, NaTFA and matrix in THF. The matrices used were 2,5-dihydroxybenzoic acid (DHB) and trans-2-[3-(4-tert-butylphenyl)-2-methyl-2-propenylidene] malononitrile (DCTB).

#### 2.2.3. Gel Curing

Gels were cured from solutions of thiol functional PEG, alkene-functional dendrimers and LAP by irradiation for 10 min with a 100 W Hg table-top UV lamp (Blak-Ray B-100AP) with a 365 nm band pass filter. Thiol functional PEG was dissolved in half the total desired solution volume of deionized water containing 1 wt.% LAP. Alkene-functional dendrimer was dissolved in an equal volume of ethanol (abs.). The dendrimer solution was then added to the PEG-solution. The mixture was ultrasonicated for 10 s and vortexed before curing.

#### 2.2.4. Rheology

Rheology tests were performed on a Discovery Hybrid Rheometer 2 from TA Instruments. Sample solutions were loaded onto the instrument, which was fitted with a 20 mm geometry and a UV LED accessory with peak emission at 365 nm. Measurements were conducted in oscillation fast sampling mode at 2% strain, with a 500 µm gap height and a frequency of 1 Hz for 60 s. The procedure was initiated and run for 10 s at which point the UV attachment was powered on at 10% power.

#### 2.2.5. Cell Experiments

Human dermal fibroblast (hDF) and mouse monocyte (Raw 264.7) cell lines were used for the cytotoxicity assays of the dendrimers and hydrogels. Cells were harvested and transferred into 96-well plates with a density of 5000 cells/well and incubated for 24 h before use. Dendrimer and polymer samples were dissolved in fresh complete DMEM (insoluble samples in DMSO) and added to the cell culture at the designed concentrations. After 24 or 72 h, 10 µL of Alamar blue agent was added into each well and the fluorescent intensity was recorded after 4 h by a plate reader (Infinite^®^ M200 (Tecan, Männedorf, Switzerland)). For the elution test of the gels, the sterilized gels were submerged in 2 mL of medium for 2 h to obtain the elution medium. One-hundred microlitres of 1%, 10%, or 100% of elution medium were then transferred into each well as the working medium to replace old medium. Alamar blue was added after 24 or 72 h as previously described. In all cases, six replicate wells were set for each sample and cells treated with PBS were used as negative control.

To observe cells in contact with the gels, the sterilized gels were placed on the bottom of the wells of a 6-well plate and 2 mL of DMEM containing 20,000 cells were applied on top of the gels. After 24 h of incubation, the medium was removed and the gels were washed twice with PBS. The plates were then observed under a microscope. 

#### 2.2.6. Bacterial Experiments

Amino and hydroxyl terminated hydrogels formed from 20 µL of curing solution were prepared for inhibition zone tests by submerging them in 2 mL of deionized water for 2 h three times to leach out unreacted constituents that could give false positives. For comparison, spherical discs with a diameter of approximately 0.9 cm were cut out of a commercial band-aid (SalvequickMED Antibact Maxi Cover) containing 0.2% of the antibacterial polymer polyhexamethylene biguanide (PHMB), which were used in the same assay. The deionized water was exchanged for PBS before the test. *E. coli* 178 and *S. aureus* 2569, as typical gram-negative and gram-positive strains, were used in the test. The cylinder-shaped hydrogels were put on MHB Ⅱ agar containing bacteria (concentration roughly 1 × 10^7^ CFU/mL). The plates were cultured at 37 °C overnight and the diameters of the inhibition zones were measured. All measurements were performed in triplicates; however, observations of the inhibition zone around the amino terminated hydrogel in *S. aureus* were based on only two of the three gels due to damage to the agar surrounding one of the gels.

#### 2.2.7. Swelling

To investigate the effect of varying the PEG length, the swelling of the hydrogels made with 2, 6 and 10 kDa PEG was studied in pH 7.4 McIlvaine’s phosphate-citrate buffer at room temperature. To investigate the effect of the swelling media and temperature, the swelling of the 10 kDa gels were studied in McIlvaine’s buffers of pH 7.4 (blood pH) and pH 5 (skin pH) and in 0.9% saline at both 37 °C and room temperature. Thirty microlitre droplets of 20% dry weight solutions of PEG, dendrimer and LAP were carefully placed on a flat PTFE surface and cured. The gels were transferred to vials and 1 mL of deionized water or buffer was added. At designated time intervals, the buffer solution was removed from the gels and their masses were recorded. All gels were tested in triplicate. The buffer/water content (WC) and swelling ratio (SR) were calculated with Equations (1) and (2):

WC = (W − W_d_)/W (1)

SR = W/W_d_ (2)

where WC is the water or buffer content, W is the weight of the swollen gel and W_d_ is the weight of the solid part of the gel, which was determined from the dry weight of the gels and the gel fraction that was measured according to the gel fraction test.

#### 2.2.8. Gel Fraction

Three 150 µL solutions of 20 wt.% gels with 10 kDa PEG were cured in 1.5 mL Eppendorf vials. The vials were cut open and the gels were transferred to glass vials. After 105 min in a 50 °C vacuum oven and 10 min in a 150 °C oven, the mass of the gels remained stable and they were assumed to be completely dry. The gels were then submerged in 5 mL of deionized water for 2 h, after which the water was exchanged and the gels were submerged for another 2 h. The process was repeated with methanol, after which the gels were again kept for 105 min in a 50 °C vacuum oven and 10 min in a 150 °C oven. Mass was again recorded and compared to the mass before leaching the gels with water and methanol.

## 3. Results and Discussion

### 3.1. Synthesis of Dendrimers and PEGs

**Scheme 1:**
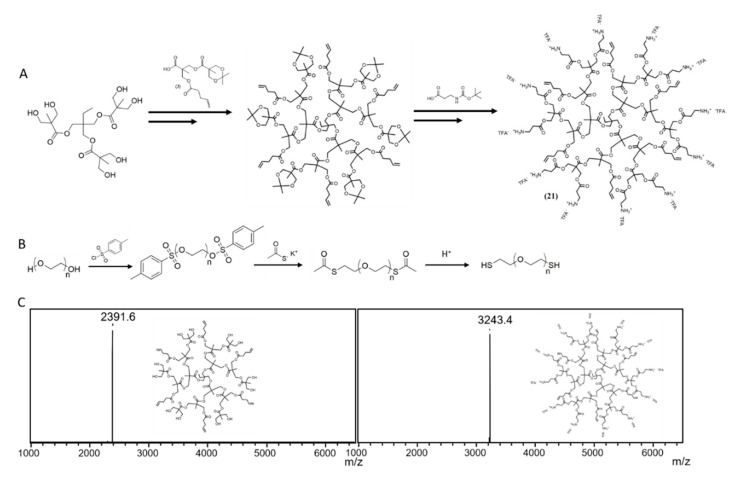
Schematic illustration of the synthesis route for (**A**) bifunctional dendrimers with alkenes and ammonium groups; (**B**) dithiol-functional PEG; and (**C**) MALDI spectra of hydroxy and alkene functional dendrimers (left) and bifunctional dendrimers with alkene and amine functionalities (right).

In a recent paper, we presented the synthesis of AB_2_C-monomers based on bis-MPA, which enabled the facile synthesis of heterofunctional polyester dendrimers via a divergent growth approach [27]. These monomers facilitate the synthesis of dendrimers, which exhibit both hydroxyls and other functional groups commonly used for cross-linking reactions such as double and triple bonds and azides. The synthetic routes for the ammonium functional dendrimer and thiol functional PEGs are illustrated in Scheme 1. The alkene-functional version of the AB_2_C monomer architecture enabled the straightforward conversion of the first-generation hydroxyl functional dendrimer into the second generation bis-MPA dendrimer with 12 acetonide protected hydroxyl groups and six alkenes for cross-linking through thiol-ene chemistry. This was carried out via fluoride promoted esterification (FPE) chemistry with imidazole-activation of the AB_2_C-monomer [17]. This FPE technique is capable of quantitatively functionalizing hydroxyl groups of even higher generation dendrimers. The imidazole by-product, excess carboxylic acid and catalytic cesium fluoride, were easily removed by washing the reaction mixture with acid and basic aqueous solutions, giving monodisperse dendrimers in high purity with minimal effort in comparison to techniques such as anhydride esterification, where removal of the urea by-product from the anhydride formation can require tedious purification techniques such as silica gel column chromatography. 

After conversion of the acetonides to hydroxyls by mild acidic catalysis in methanol, the 12 hydroxyl groups on this dendrimer were reacted with boc-protected β-alanine—again with FPE chemistry, as shown in Scheme 1. The boc-groups were then removed by dissolving the dendrimer in a mixture of trifluoroacetic acid (TFA) and chloroform, resulting in monodisperse dendrimers with 12 ammonium groups and six alkenes. Three reaction steps were required to synthesize these dendrimers from commercially available first generation bis-MPA dendrimers and the previously synthesized AB_2_C-monomer, which was made in four reaction steps and had a total yield of 55%. The characteristic monodispersity of dendrimers was confirmed for these materials by MALDI-TOF MS, as only ion adducts of the expected products were detected without structural flaws. Such spectra are demonstrated in Scheme 1 for the hydroxyl and amino-functional dendrimers.

Dithiol PEGs were synthesized through tosylation of the hydroxyls followed by substitution of the tosylate with thioacetate, as shown in Scheme 1. The thioacetate PEGs were then converted into thiol functional PEGs through protonation with an acid. This route was used as opposed to the faster route of connecting an acid, such as mercaptopropionic acid, to the PEGs in order to create a network that would be more stable in solution, with only one ester connecting the PEG to the dendrimer. The procedure was carried out successfully on commercial PEGs with average molecular weights of 2, 6 and 10 kDa. MALDI-MS, ^1^H and ^13^C NMR spectra and detailed protocols for the synthesis of the thiol functional PEGs are provided in the supporting information.

### 3.2. Formulation and Curing of Hydrogels

**Figure 1:**
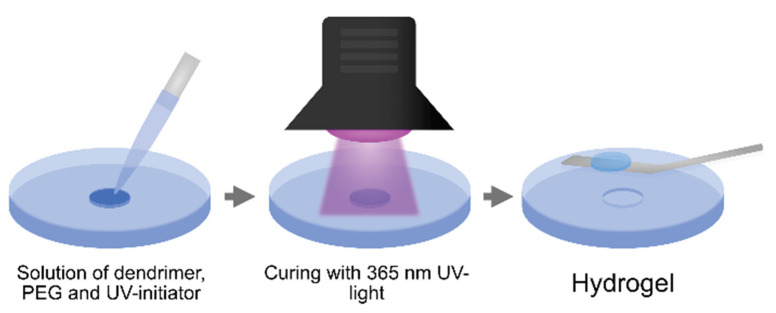
Functional dendritic hydrogels were formed from solutions of dendrimer, dithiol-functional PEG and photoinitiator in equal parts ethanol and water. The solutions were cured under a UV-lamp with peak emission at 365 nm.

In all network formulations, a stoichiometric ratio of thiols to alkenes was used—i.e., a 1:3 molar ratio of dendrimer to PEG. The solutions solidified within 10 min of irradiating the mixture with both 10 and 20 dry wt.% solutions with 0.5 wt.% of LAP as photoinitiator. This simple formulation process is illustrated in Figure 1. The networks formed from the 10 wt.% solution were found to be mechanically fragile, while the 20 wt.% gel could be extracted from the curing mold, swelled with water and handled without breaking. This result led to the 20 dry wt.% solution being used for all future hydrogels throughout this work.

In addition to the 2, 6 and 10 kDa PEGs, a monomethylated 5 kDa PEG was also thiolated and reacted with the dendrimer under the same conditions as the hydrogel curing in order to study the efficiency of the thiol-ene reaction using NMR spectroscopy. Within 15 min of irradiation, the double bonds could no longer be observed in the ^1^H NMR spectrum (Figures S2–S4), which highlighted the high efficiency of the reaction as the concentration of thiols and alkenes was only 19 mM in the curing solutions.

**Figure 2:**
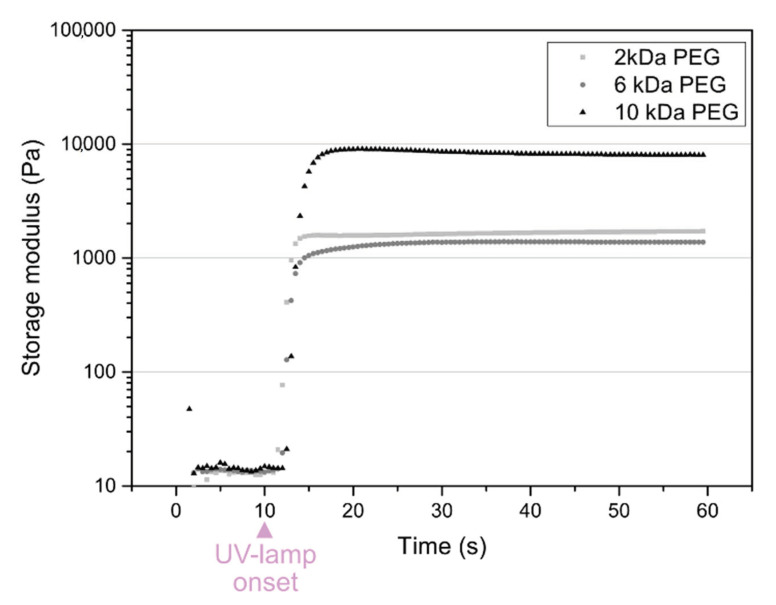
Hydrogels were cured on a circular rheometer geometry equipped with a UV-lamp while monitoring the modulus. The storage modulus stabilized within seconds of the onset of the UV-lamp and the highest modulus after curing was recorded for the 10 kDa gels.

The curing of the networks was analysed in real-time by monitoring the change in modulus while curing the networks within a rheometer. Solutions of the 2, 6 and 10 kDA thiol-functional PEG and dendrimer were added to a circular rheometer geometry and a UV-lamp was turned on after 10 s of oscillation, forming a thin film. As Figure 2 shows, the modulus rapidly increased and stabilized within 10 s regardless of the PEG size, as a result of the high reaction rate of the thiol-ene reaction.

The storage modulus of the 10 kDa PEG gel directly after curing was approximately 8 kPa while the modulus of the 2 k and 6 k PEG gels were 1.7 and 1.4 kPa respectively. In general, a network with a short PEG is stiffer due to the increased crosslinking density and shorter chain segments between the cross-links [27,28]. In order to maintain stoichiometric equilibrium of thiols and alkenes, the PEG to dendrimer weight ratio increased from 1.3:1 to 3.9:1 to 6.5:1 for the gels with 2 kDa, 6 kDa and 10 kDa PEG, respectively. Even though the shortest PEG potentially would give the stiffest network, the gel had a higher mass content of dendrimer compared to PEG, which greatly influenced its mechanical properties. It is also possible that the longest PEG had a higher capability of forming loops between the chains in the network, which would have contributed to its stiffness by limiting chain movements. The shorter PEGs should statistically have also had a higher probability of reacting through both terminal thiol groups with the same dendrimer. Such bidentate reactions would have reduced the final cross-linking density and resulted in a lower modulus. 

### 3.3. Swelling of Hydrogels

**Figure 3:**
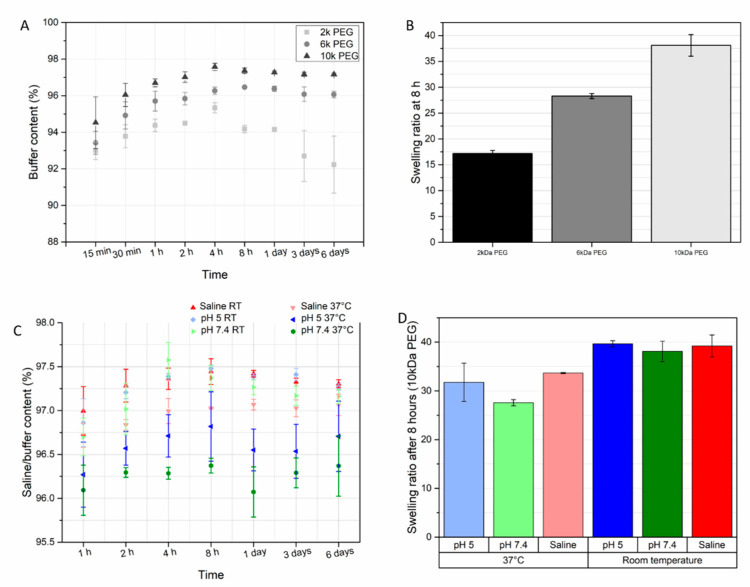
(**A**) The swelling of hydrogels formed with different dithiol functional PEGs in citrate-phosphate buffer (pH 7.4) at room temperature (RT); (**B**) the swelling ratios of the hydrogels after submersion in the buffer for 8 h at RT. (**C**) the swelling of the hydrogels formed with 10 kDa PEG at different pH and temperature; (**D**) the swelling ratios of hydrogels formed with 10 kDa PEG at different pH and temperature.

The effect of the PEG length on swelling was studied by measuring the swelling of gels made with 2, 6 and 10 kDa PEG in a pH 7.4 phosphate-citrate buffer at room temperature. The mass of the 6 and 10 kDa PEG gels stabilized after approximately 4 h. The highest mass of the 2 kDa gels was also recorded after 4 h; however, this gel started to disintegrate after this measurement, which led to a significant decrease in recorded mass at 8 h and upwards. These swelling results are presented in Figure 3A.

The swelling ratios of all three gels were significantly different at both 4 and 8 h (Figure 3B). At these times, the 10 kDa gels averaged a solid content of 2–3%, which corresponded to a swelling ratio of around 40. The corresponding values for the 6 kDa gels were around 3–4%, with swelling ratios slightly below 30. The 2 kDa gel showed the least amount of swelling, with solution contents of 5–6% at most and swelling ratios only reaching slightly above 20. Therefore, the extent of swelling increased with increasing PEG size, which was probably due to the inverse relationship between PEG size and crosslinking density. A more sparsely crosslinked gel should have more free space for water to reside in, which is a logical explanation to the results and in line with previously studied dextran hydrogels [29].

The 10 kDa gel was able to absorb around 40 times its dry weight in pH 7.4 buffer at room temperature and a fully swollen gel only contained around 0.3% of the valuable dendrimer. In other words, only a few milligrams of dendrimer were required to make 1 g of fully swollen gel, which in combination with its higher modulus and strength made it the most interesting of the three gels. To further investigate the influence of the swelling media and temperature on the swelling capability of the 10 kDa gel, it was studied in 0.9% saline, pH 5 (approximate skin pH) and pH 7.4 (blood pH) at room temperature and 37 °C. The results are shown in Figure 3C,D. This study showed that the temperature seemed to influence the swelling ratio more than salt concentration or pH, as the swelling was significantly lower at 37 °C than at room temperature, while insignificantly different for the different solutions at the same temperature.

A high degree of swelling is favourable for many different applications, as it allows for the gel to be loaded with more of a drug or a liquid of interest. A high degree of swelling can also be a desired property in a wound dressing, which should absorb fluid exuded from the wound while maintaining a moist atmosphere to facilitate healing [30]. High water content is also favourable for oxygen transmission, since oxygen typically diffuses better in water than in the gel part of a hydrogel.

### 3.4. Post-Functionalization of Hydrogels with the NHS-Ester of Disperse Red 13

The amino groups on the dendritic component in these gels were not involved in the hydrogel formation. Amino groups are highly interesting when it comes to functionalization reactions, since they are strong nucleophiles. Reacting amines with esters of N-hydroxysuccinimide at neutral to basic pH to give amides is a well-established method. Amines are also cationically charged at neutral to low pH and can interact electrostatically with anionic molecules. To investigate whether the gels could be covalently functionalized with NHS-esters and electrostatically loaded with anionic molecules, disperse red 13 was used as a model compound since its deep red colour is a clear indicator of its presence or absence.

**Figure 4:**
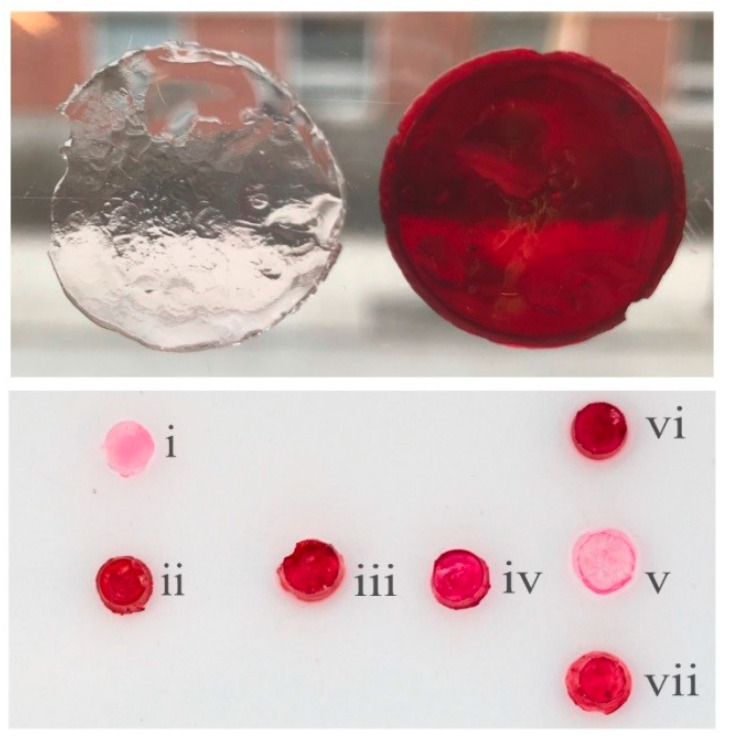
The top image shows a gel made with hydroxyl-functional dendrimers and 10 kDa PEG (left) and amino-functional dendrimers and the same PEG (right). Both gels have been treated with the same amount of NHS-activated dye and washed equal times with solutions of methanol and water. The bottom image shows gels treated with the dye after 8 h (iii), 24 h (iv) and 48 h (v) in pH 7.4 buffers at 37 °C and after 48 h in pH 4.4 at 37 °C (vi) and 48 h in pH 7.4 at room temperature (vii). (i) and (ii) are hydroxyl and amino-functional gels subjected to the dye directly after curing for reference.

Figure 4 shows hydroxyl and amino-functional gels that have been reacted with the NHS-ester of disperse red 13 in a mixture of pH 7.4 buffer and methanol, followed by extensive washing to remove any unreacted dye. The covalent functionalization with the NHS ester seemed to be highly efficient, as was evident from the clear difference in colour between the two gels. This result was also supported by studying a solution of the free hydroxyl and amino-functional dendrimers with the NHS-ester in the same buffer and methanol solution in MALDI-MS, where full conversion of the amino-groups could be detected within an hour of reaction time while the hydroxyl-functional dendrimers remaining unaffected (data not shown).

To investigate whether the degradation of the gels proceeded similarly to the free dendrimers in solution, the gels were kept in buffer solutions at pH 7.4 in 37 °C. Under these conditions, the free amino-functional dendrimers showed rapid degradation as their β-alanine end-groups detach, exposing the hydroxyl groups and a similar degradation mechanism should most likely be expected within the hydrogels.

After 8, 24 and 48 h, the gels were coloured with the NHS-ester of disperse red 13, which had proven to be reactive specifically towards the amino-groups on the dendrimers in the gels. After washing away unreacted dye, the remaining colour of the gels gave a qualitative indication of the number of primary amines present in the gels when compared to newly prepared amino functional gels, which had not been post-functionalized with disperse red 13. The free amino-functional dendrimers degraded through loss of β-alanine end-groups and this test indicated a very similar behaviour, albeit at a slower rate, to the dendrimers bound in the gel, as shown in Figure 4. After being submerged in pH 7.4 buffer for 48 h at 37 °C, the 10 kDa gels showed no visual signs of physical degradation. However, the intensity of the red colour was significantly lower than that of the freshly prepared gel after having added the dye. The lighter red colour indicated that these gels contained a lower concentration of disperse red, which suggested that the β-alanine groups had been removed from the dendritic component of the gels by hydrolysis.

### 3.5. Biocompatibility and Antibacterial Activity of Hydrogels

**Figure 5:**
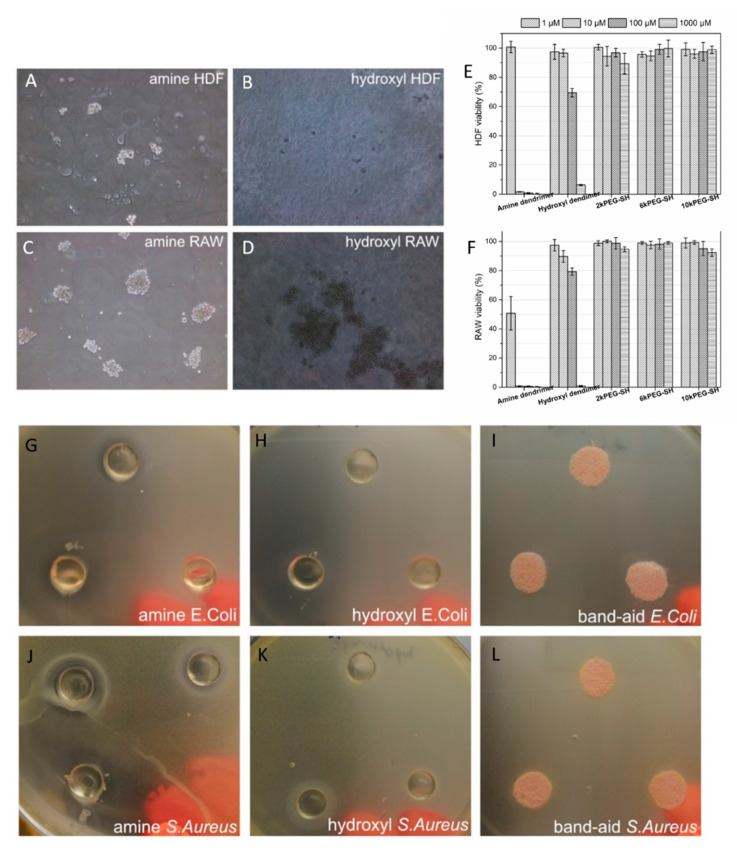
(**A**–**D**) The effects of gels on HDF and RAW 264.7 when cells were in direct contact with gels; (**E**,**F**)) cytotoxicity of the dendrimers and thiol-terminated crosslinkers towards HDF and RAW 264.7 determined by Alarmarblue assay after 24 h incubation; and (**G**–**L**)) antibacterial activity of the amine, hydroxyl gels and commercial band-aid against *E. coli* and *S. aureus*. The agar surrounding one of the gel samples in J was damaged, but inhibition zones are clearly visible around the other two amino-containing hydrogels in *S. aureus*.

The biocompatibility of the dendrimers and hydrogels was evaluated against human dermal fibroblasts (HDF) and monocytes (RAW 264.7). Cells were cultured with different concentrations of the difunctional dendrimers and the thiol-functional PEGs. The hydrogels were submerged in 2 mL cell culture medium to obtain the elution medium in order to see if hydrogel leach out was toxic towards cells. These results are presented in Figure 5. The heterofunctional dendrimers containing cationic amine groups were found to be toxic towards both cells even at lower concentration of 10 µM (Figure 5E,F). The amphiphilic nature of these dendrimers was expected to cause cytotoxicity, since amphiphiles are known to interact strongly with cell membranes and in some cases disrupt them [31,32]. The hydroxyl functional dendrimers were, however, better tolerated by both cell lines, with cell viability above 70% at concentrations ranging from 1–100 µM. Hydroxyl functional materials are in general more benign than amino or ammonium functional counterparts and the amphiphilicity of the hydroxyl and alkene-functional dendrimers is lower since the hydroxyls are less hydrophilic than amines. The thiol functional PEG crosslinkers did not exhibit significant toxicity even at the highest tested concentration (1 mM) with cell viability of 90%. This could be a concern for clinical applications if the difunctional dendrimers are not quantitatively incorporated in the cured network. To determine the gel fraction—the actual weight of the dry gels compared to the added dry material before curing—gels were fully dried and treated with water and methanol to leech out components that were not covalently incorporated into the gel network and dried again. The three gels averaged a remaining solid content of 82.6% with a standard deviation of 4.3% after leaching out non-gelled constituents. This is an acceptable number in comparison to previously reported materials, but still stresses the importance of investigating the effect of any constituents that could leach out of the cross-linked material. 

Interestingly, no significant cell death was detected when cells were cultured with non-diluted 100% hydrogel leach out medium (Figure S24) and both amine gel and hydroxyl gel showed cell viability above 95%. This indicates that a very low number of unreacted dendrimers was leaching out of the hydrogels and a large majority of the dendrimers was incorporated into the network, or incorporated into smaller networks that were not connected to the large network making up the hydrogels. In another assay, cells were co-cultured with the presence of gels. Unfortunately, the results were more difficult to interpret and quantify. Attempts to quantify the amount of cell death were deemed inaccurate since the vast majority of cells were able to migrate away from the gel and the cells residing on the gels were instead inspected visually through a microscope (Figure 5A–D). Both HDF and RAW cells were capable of proliferating on the surface of the ammonium-functional 10 kPEG gels. The HDF cells residing on the gel surface were quite constrained and circular, however, they were alive. This indicated that, while extensive cell death was not induced, the cells were not content with the environment. The monocytes, however, proliferated to a higher degree on the gel surfaces in clusters and did not differentiate into macrophages, which is their natural response when in contact with a foreign object. These results, however qualitative, are very promising and point towards these materials being biocompatible. For comparison, cells were cultured in the presence of hydroxyl-functional gels formed from 10kPEG and dendrimer **3**. Very similar results were obtained, indicating that the ammonium groups do not seem to significantly influence the cells when they are residing on the surface of the gel.

From the swelling experiments, it could be deduced that the dendrimer concentration was approximately 650 µM for the 10 kDa PEG gels at maximum swelling. Complete cell death in the presence of free dendrimer was detected already at 10 µM and the lack of toxicity from the leach out medium is therefore quite surprising. This could be a result of the large number of functional groups on each dendrimer available for cross-linking. As each dendrimer had six double bonds that could react with the PEGs, the probability that a dendrimer was not connected to a larger network should have been low. As the double bonds are converted into thio-ether linkages to the PEGs, the amphiphilicity that largely contributes to the cytotoxicity is reduced.

Since the hydrogels contained cationic amine groups, which are supposed to have a strong interaction with the anionic surface of the bacterial membrane, the disk diffusion test was used to assess the antibacterial activity of the hydrogels against both gram-negative *E. coli* and gram-positive *S. aureus*. The hydrogels were put on the agar plates containing bacterial concentrations of approximately 10^7^ CFU/mL. After overnight incubation, the agar plates were taken out to see whether the hydrogels had any effect on the bacteria. The images are shown in Figure 5. There were small inhibition zones around the amino gels towards both *E. coli* (Figure 5G) and *S. aureus* (Figure 5J), with an average diameter of 1.4 cm and 1.3 cm, respectively. These experiments were performed in triplicate; however, observations were made from only two of the three amino containing hydrogel samples in *S. aureus* due to damage to the agar surrounding one of the gels (Figure 5J). The inhibition zones suggested that the leaching out of the gel can inhibit the growth of bacteria. The hydrogels were shown to rapidly lose their amino-groups under physiologically conditions, meaning that the gels will degrade with time. This is a highly desired property, because the antibacterial gel fragments will not accumulate in the environment and will therefore prevent the development of antibiotic resistance. 

The hydroxyl gels had no effect on either bacterial growth, as there was no inhibition zone on the agar plates (Figure 5H,K). The commercial antibacterial wound dressing (SalvequickMED Antibact) containing an antibacterial cationic polymer showed limited inhibition effect on bacterial growth; however, only the bacterial density under the patch was decreased. The molecular weight of the polyhexamethylene biguanide (PHMB) that the band-aid was impregnated with was not stated by the manufacturer. There are however reports stating that commercially available PHMB is a mix of oligomers with around 12 repeating units and a molecular weight of around 2400 g/mol [33]. If that is the case, this would result in a total number of cations in the band-aid impregnated with 0.2% PHMB of around 10 µmol/g. The same number for the amino-functional hydrogels would be around 8 µmol/g considering complete protonation of all amines on every dendrimer, which would not be the case at pH 7.4. The two can thus be considered to be comparable in this study, since the surface areas of the materials that were in contact with the bacteria were very similar. In conclusion, the hydrogels formulated with amino-functional bis-MPA dendrimers and 10 kDa PEG appeared to be more efficient at inhibiting the growth of both gram-positive (*S. aureus*) and gram-negative (*E. coli*) bacteria than gels formulated with hydroxyl-functional dendrimers and a commercially available antibacterial band-aid.

## 4. Conclusions

Heterofunctional bis-MPA dendrimers with 12 ammonium trifluoroacetate and six alkene groups were synthesized and used together with thiol-functional PEGs made from commercially available 2, 6 and 10 kDa PEG to form cross-linked networks. The networks were cured under 365 nm UV-light using LAP as a photoinitiator. The networks absorbed aqueous solutions rapidly and gels made with 10 kDa PEG absorbed 40 times their dry weight in pH 7.4 phosphate-citrate buffer within 4 h. In general, swelling increased with increasing PEG length. The gel containing 10 kDa PEG had the highest storage modulus after curing of around 8 kPa and was also the strongest gel that could withstand the most amount of physical manipulation without breaking.

The amino-groups of the gel proved to be highly accessible for post-functionalization after gelation through NHS-chemistry since the gels could be functionalized with NHS-activated disperse red 13, resulting in the gels having a strong red color.

The gels were structurally stable after having been submerged in a phosphate-citrate buffer of pH 7.4 and kept at 37 °C for up to 48 h while the dendrimers in the networks lost their β-alanines under these conditions through hydrolysis in the same manner as the previously reported amino-functional dendrimers based on bis-MPA. This enabled the materials to make use of the functionality of the amino groups while degrading into safer hydroxyl functional materials with time, reducing antibacterial waste and the release of antibacterial constituents throughout the body.

HDF and monocytes were cultured on top of the hydrogels and seemed to tolerate the materials well. Meanwhile, the gels made with amino-functional dendrimers clearly inhibited the growth of both gram-positive and gram-negative bacteria, while gels made from the same dendrimers but with hydroxyl groups showed no reduction of bacterial growth at all.

We believe that these materials demonstrate the antibacterial effect of cationic polyester-based macromolecules and that they are an interesting addition to the constantly growing family of highly advanced antibacterial materials with potential for high biocompatibility with potential applications as wound-dressings or coatings with further development.

## Supplementary Materials

More detailed material synthesis and MALDI-MS and NMR spectroscopy characterizations of the materials is available online.

## Sample Availability

Samples of the compounds may be available from the authors.
