# Supplementary material for: UV-Cured Antibacterial Hydrogels Based on PEG and Monodisperse Heterofunctional Bis-MPA Dendrimers
Source: Molecules. 2021 Apr 19;26(8):2364. doi: 10.3390/molecules26082364 (PMC8073803; doi:10.3390/molecules26082364)
Supplement: Supplementary file 1 [file molecules-26-02364-s001.zip › molecules-1173236-supplementary/molecules-1173236-supplementary.pdf]

Supporting Information

UV-cured functional hydrogels based on PEG and monodisperse bis-MPA dendrimers with antibacterial properties

Patrik Stenström [1], Yanmiao Fan [1], Yuning Zhang [1], Daniel Hutchinson [1], Sandra García-Gallego [2], Michael Malkoch [1,*]

[1] Department of Fibre and Polymer Technology, Division of Coating Technology, KTH Royal Institute of Technology, Teknikringen 48, 114 28 Stockholm, Sweden; malkoch@kth.se

[2] Department of Organic and Inorganic Chemistry, University of Alcalá, 28871 Madrid, Spain; Sandra.garciagallego@uah.es

* Correspondence: malkoch@kth.se

1. Materials and Methods

1.1. Chemicals

Bis-MPA was obtained in kind from Perstorp AB. The first-generation hydroxyl functional dendrimer was synthesized according to a previously published procedure [1]. PEG2k, PEG6k, PEG10k and mPEG5k were purchased from Sigma Aldrich. CDI was purchased from Carbosynth. Cesium fluoride was purchased from Sigma Aldrich. 4-pentenoic acid was purchased from Sigma Aldrich. Boc-protected beta-alanine was prepared according to previously published procedures [2,3]. Deuterated solvents were purchased from Cambridge Isotope Laboratories. Ethyl acetate, heptane, methanol, palladium on carbon, butanol, benzyl alcohol, DHB and DCTB for MALDI were purchased from Sigma Aldrich. DMEM, PBS, penicillin/ streptomycin and tripsin-EDTA were purchased from Gibco. Human Dermal Fibroblasts (HDF) and mouse monocytes (RAW 264.7) were purchased from ATCC (American Tissue Culture Collection) and maintained in Dulbecco's Modified Eagle Medium (DMEM) containing 10 % fetal bovine serum FBS, penicillin G (100 IU) and streptomycin 100 mg/L streptomycin under 5% CO_2 at 37 °C. SalvequickMED Antibact Maxi Cover was purchased from Apotea AB, Sweden.

1.2. NMR Spectroscopy

^1H and ^{13}C NMR spectroscopy were performed on a 400 MHz Bruker Avance III. ^1H NMR spectroscopy was performed using 32 scans, 1 s relaxation delay and 20 ppm spectral window. The respective values for ^{13}C NMR spectroscopy were 512 scans, 2 s and 240 ppm. Samples were analyzed in deuterated chloroform or deuterated methanol. The spectra were analyzed with MestreNova v. 9.0.0-12821 from Mestrelab Research.

1.3. MALDI-MS

MALDI-MS was carried out using a Bruker Ultraflex-III. The instrument was calibrated using dendritic calibrants from Polymer Factory AB, Stockholm, Sweden. Samples were prepared using a mass ratio of

1:1:40 of sample, NaTFA, and matrix in THF. Matrices used were *trans*-2-[3-(4-tert-Butylphenyl)-2-methyl-2-propenylidene]malononitrile (DCTB) and 2,5-dihydroxybenzoic acid (DHB).

1.4. Gel curing

Gels were cured from solutions of thiol functional PEG, alkene-functional dendrimers and LAP by irradiation for 10 minutes with a 100W Hg table-top UV lamp (Blak-Ray B-100AP) 365 nm band pass filter. Thiol functional PEG was dissolved in half the total desired solution volume of deionized water containing 1 wt.% LAP. Alkene-functional dendrimer was dissolved in an equal volume of ethanol (abs.). The dendrimer solution was then added to the PEG-solution. The mixture was ultrasonicated for 10 seconds and vortexed before curing.

1.5. Rheology

Rheology tests were performed on a Discovery Hybrid Rheometer 2 from TA Instruments. Sample solutions were loaded onto the instrument, which was fitted with a 20 mm geometry and a UV LED accessory with peak emission at 365 nm. Measurements were conducted in oscillation fast sampling mode at 2% strain, 500 μm gap height, and a frequency of 1 Hz for 60 seconds. The procedure was initiated and run for 10 seconds at which point the UV attachment was powered on at 10% power.

1.6. Cell experiments

Cytotoxicity tests of dendrimers, polymers and gels were measured with the Alamar blue assay. Cells were harvested and transferred into 96 well plates with a density of 5000 cells/well and incubated for 24 h before use. Dendrimer and polymer samples were dissolved in fresh complete DMEM (insoluble samples in DMSO) and added to the cell culture at the designed concentrations. After 24 or 72 hours, 10 μL of Alamar blue agent was added into each well and the fluorescent intensity was recorded after 4 h by a plate reader (Infinite® M200 (Tecan, Switzerland)). For the elution test of the gels, the sterilized gels were submerged in 2 mL of medium for 2 hours to obtain the elution medium. 100 μL of 1%, 10% or 100% of elution medium were then transferred into each well as the working medium to replace old medium. Alamar blue was added after 24 or 72 hours as previously described. In all cases, 6 replicate wells were set for each sample and cells treated with PBS were used as negative control.

To observe cells in contact with the gels, the sterilized gels were placed on the bottom of the wells of a 6 well plate and 2 mL of DMEM containing 20,000 cells were applied on top of the gels. After 24 hours of incubation, the medium was removed and the gels were washed twice with PBS. The plates were then observed under a microscope.

1.7. Bacterial experiments

Amino and hydroxyl terminated hydrogels formed from 20 μL of curing solution were prepared for the inhibition zone tests by submerging them in 2 mL of deionized water for 2 hours three times to leach out unreacted constituents that could give false positives. For comparison, spherical discs with a diameter of approximately 0.9 cm were cut out of a commercial band-aid (SalvequickMED Antibact Maxi Cover) containing 0.2% of the antibacterial polymer polyhexamethylene biguanide (PHMB), which were used in the same assay. The deionized water was exchanged for PBS before the test. E. coli 178 and S. aureus 2569 were used in the test as typical gram-negative and gram-positive strains. The cylinder-shaped hydrogels were put on MHB II agar containing bacteria (concentration approximately

1×107 CFU/mL). The plates were cultured at 37 °C overnight and the diameters of the inhibition zone were measured. All measurements were performed in triplicates.

1.8. Swelling

To investigate the effect of varying the PEG length, the swelling of the hydrogels made with 2, 6 and 10 kDa PEG was studied in pH 7.4 McIlvaine's phosphate-citrate buffer at room temperature. To investigate the effect on the swelling media and temperature, the swelling of the 10 kDa gels was studied in McIlvaine's buffers of pH 7.4 (blood pH) and pH 5 (skin pH), and in 0.9 % saline at both 37°C and room temperature. 30 μL droplets of 20 % dry weight solutions of PEG, dendrimer and LAP were carefully placed on a flat PTFE surface and cured. The gels were transferred to vials and 1 mL of deionized water or buffer was added. At designated time intervals, the buffer solution was removed from the gels and their mass was recorded. All gels were tested in triplicate. The buffer/water content and swelling ratio was calculated with equations 1 and 2:

$$WC = (W - W_d) / W \quad (1)$$

$$SR = W / W_d \quad (2)$$

where *WC* is the water or buffer content, *W* is the weight of the swollen gel and W_d is the weight of the solid part of the gel, which was determined from the dry weight of the gels and the gel fraction that was measured according to the gel fraction test.

1.9. Gel fraction

Three 150 μL portions of 20 wt.% gels with 10kDa PEG were cured from 150 μL solutions in 1.5 mL Eppendorf vials. The vials were cut open and the gels were transferred to glass vials. After 105 minutes in a 50°C vacuum oven and 10 minutes in a 150°C oven, the mass of the gels remained stable and they were assumed to be completely dry. The gels were then submerged in 5 mL of deionized water for 2 hours, after which the water was exchanged and the gels were submerged for another two hours. The process was repeated with methanol, after which the gels were again kept for 105 minutes in a 50°C vacuum oven and 10 minutes in a 150°C oven. Mass was again recorded and compared to the mass before leaching the gels with water and methanol.

2. Synthesis procedures

*2.1. Synthesis of acetonide protected bis-MPA (**1**)*

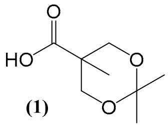

(1)

This product was synthesized according to a previously published procedure [4]. The product was also recrystallized in acetone to increase the purity.

*2.2. Synthesis of benzyl protected bis-MPA (**2**)*

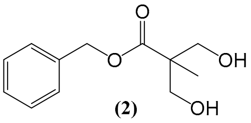

(2)

This product was synthesized according to a previously published procedure [4].

*2.3. Synthesis of AB$_2$C-ene monomer (**3**)*

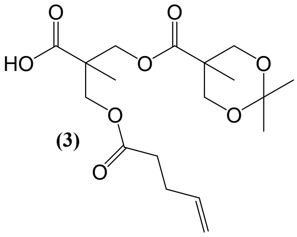

(3)

This product was synthesized according to a previously published procedure [5].

*2.4. Synthesis of PEG-2k-OTs (**4**)*

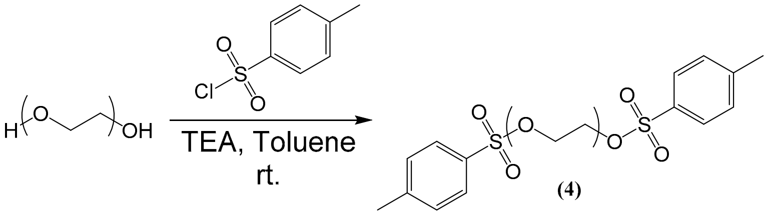

(4)

25 g (12.5 mmol, 1 eq) of 2 kDa PEG was dissolved in 100 mL of toluene and 30 mL of TEA (250 mmol, 20 eq) at 45°C. 35.8 g (188 mmol, 15 eq) of tosyl chloride was added, and the reaction was carried out for 14 h at 45°C. The reaction mixture was then diluted with 500 mL of toluene in an extraction funnel and allowed to cool to room temperature before being washed with 4 x 125 mL of deionized water. The organic phase was dried with $MgSO_4$ filtered and rotary evaporated. The resulting oil was dissolved in dichloromethane and precipitated in ether three times, after which 4.2 g (1.92 mmol, 0.15 eq) of product was obtained as an off-white powder.

^{1}H-NMR: (400 MHz, Chloroform-*d*) δ 7.86 – 7.74 (m, 4H, **CH**=C(S)-**CH**), 7.34 (d, J = 8.0 Hz, 4H, **CH**=C(Me)-**CH**), 4.21 – 4.10 (m, 4H, **CH$_2$**-O-S), 3.64 (s, 186H, **CH$_2$**-**CH$_2$**-O), 3.58 (s, 8H, **CH$_2$**-**CH$_2$**-O), 2.44 (s, 6H, C-**CH$_3$**).

^{13}C-NMR: (101 MHz, Chloroform-*d*) δ 144.78 (**C**-S(=O)$_2$-O), 133.07 (**C**-CH$_3$), 129.84 (**C**H=C(Me)-**C**H), 127.98 (**C**H=C(S)-**CH**), 70.76, 70.59, 70.53, 69.26 & 68.69 (**C**H$_2$-**C**H$_2$-O), 21.66 (C-**C**H$_3$).

*2.5. Synthesis of PEG-2k-SAc (**5**)*

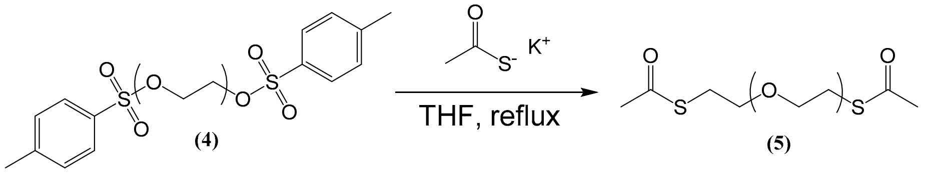

4.1 g (1.78 mmol, 1 eq) of tosylated 2 kDa PEG was dissolved in 30 mL of THF. 2.03 g (17.8 mmol, 10 eq) of potassium thioacetate was added, and the mixture was refluxed for 14 h. The resulting red/brown mixture was added to an extraction funnel along with 100 mL of deionized water, and this THF/water phase was treated with 3 x 100 mL of dichloromethane. The combined organic phases were then washed with 4 x 75 mL of 10% $NaHCO_3$ in water. The organic phase was dried with $MgSO_4$, rotary evaporated and the product was precipitated in ether. The obtained powder was dissolved in dichloromethane and precipitated again in ether to give 2.3 g (1.08 mol, 0.61 eq) of product as a slightly brown powder after filtration.

^{1}H-NMR: (400 MHz, Chloroform-*d*) δ 3.64 (s, 199H, C**H$_2$**-C**H$_2$**-O), 3.09 (t, J = 6.5 Hz, 4H, CH$_2$-C**H$_2$**-S), 2.33 (s, 6H, C(=O)-C**H$_3$**).

^{13}C-NMR: (101 MHz, Chloroform-*d*) δ 195.55 (S-**C**(=O)-CH$_3$), 70.73, 70.66, 70.60, 70.41 & 69.84 (**C**H$_2$-**C**H$_2$-O), 30.65 (CH$_2$-**C**H$_2$-S), 28.93 (S-C(=O)-**C**H$_3$).

*2.6. Synthesis of PEG-2k-SH (**6**)*

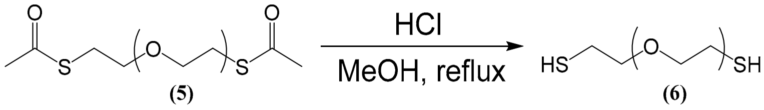

2.3 g (1.08 mmol, 1 eq) of 2 kDa thioacetate PEG was dissolved in 40 mL of methanol. 8 mL of concentrated hydrochloric acid was added, and the solution was refluxed for 2 hours. The product was precipitated in ether, dissolved in dicholoromethane and precipitated once more in ether, which gave 1.5 g (750 µmol, 0.69 eq) as an off-white powder after filtration.

^{1}H-NMR: (400 MHz, Chloroform-*d*) δ 3.64 (s, 209H, C**H$_2$**-C**H$_2$**-O), 2.69 (dt, J = 8.2, 6.4 Hz, 4H, O-CH$_2$-C**H$_2$**-SH), 1.59 (t, J = 8.2 Hz, 2H, -S**H**).

^{13}C-NMR: (101 MHz, Chloroform-*d*) δ 72.99, 70.76, 70.68 & 70.35 (**C**H$_2$-O), 24.38 (**C**H$_2$-SH).

*2.7. Synthesis of PEG-6k-OTs (**7**)*

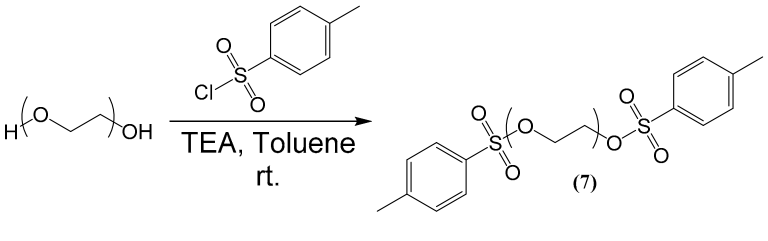

25 g (4.17 mmol, 1 eq) of 2 kDa PEG was dissolved in 100 mL of toluene and 12 mL of TEA (83.4 mmol, 20 eq) at 45°C. 11.9 g (62.5 mmol, 15 eq) of tosyl chloride was added, and the reaction was carried out for 14 hours at 45°C. The reaction mixture was then diluted with 500 mL of toluene in an extraction funnel and allowed to cool to room temperature before being washed with 4 x 125 mL of deionized water. The organic phase was dried with $MgSO_4$ filtered and rotary evaporated. The resulting oil was dissolved in dichloromethane and precipitated in ether twice, after which 16.2 g (2.62 mmol, 0.63 eq) of product was obtained as an off-white powder.

^{1}H-NMR: (400 MHz, Chloroform-*d*) δ 7.95 – 7.71 (m, 4H, C**H**=C(S)-C**H**), 7.34 (d, J = 8.0 Hz, 4H, C**H**=C(Me)-C**H**), 4.22 – 4.11 (m, 5H, $\mathbf{CH_2}$-O-S), 3.64 (s, 583H, $\mathbf{CH_2}$-$\mathbf{CH_2}$-O), 3.58 (s, 8H, $\mathbf{CH_2}$-$\mathbf{CH_2}$-O), 2.45 (s, 6H, C-$\mathbf{CH_3}$).

^{13}C-NMR: (101 MHz, Chloroform-*d*) δ 144.71 (**C**-S(=O)$_2$-O), 133.04 (**C**-CH_3), 129.79 (**C**H=C(Me)-**C**H), 127.93 (**C**H=C(S)-**C**H), 70.54, 69.21 & 68.64 (**C**H_2-**C**H_2-O), 21.60 (C-**C**H_3).

*2.8. Synthesis of PEG-6k-SAc (**8**)*

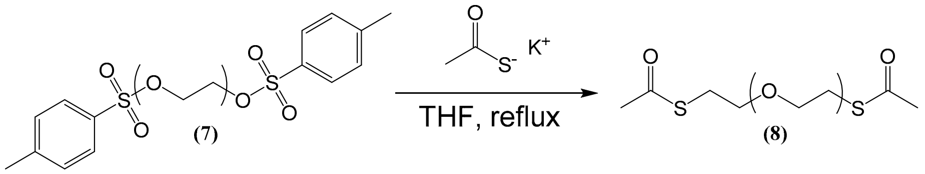

6.0 g (952 μmol, 1 eq) of tosylated 6 kDa PEG was dissolved in 20 mL of THF. 2.03 g (17.8 mmol, 10 eq) of potassium thioacetate was added, and the mixture was refluxed for 14 hours. The resulting red/brown mixture was added to an extraction funnel along with 100 mL of deionized water, and this THF/water phase was treated with 3 x 100 mL of dichloromethane. The combined organic phases were then washed with 4 x 75 mL of 10% $NaHCO_3$ in water. The organic phase was dried with $MgSO_4$, rotary evaporated and the product was precipitated in ether. The obtained powder was dissolved in dichloromethane and precipitated again in ether to give 5.0 g (819 μmol, 0.86 eq) of product as a slightly brown powder after filtration.

^{1}H-NMR: (400 MHz, Chloroform-*d*) δ 3.64 (s, 611H, $\mathbf{CH_2}$-$\mathbf{CH_2}$-O), 3.09 (t, J = 6.5 Hz, 4H, CH_2-$\mathbf{CH_2}$-S), 2.33 (s, 6H, C(=O)-$\mathbf{CH_3}$).

^{13}C-NMR: (101 MHz, Chloroform-*d*) δ 195.46 (S-**C**(=O)-CH_3), 70.66, 70.60, 70.54, 70.35 & 69.78 (**C**H_2-**C**H_2-O), 30.59 (CH_2-**C**H_2-S), 28.87 (S-C(=O)-**C**H_3).

2.9. Synthesis of PEG-6k-SH (**9**)

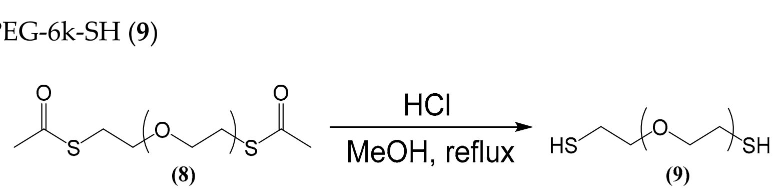

4.0 g (654 μmol, 1 eq) of 6 kDa thioacetate PEG was dissolved in 85 mL of methanol. 5 mL of concentrated hydrochloric acid was added, and the solution was refluxed for 3 hours. The product was precipitated in ether, dissolved in dicholoromethane and precipitated once more in ether, which gave 3.5 g (580 μmol, 0.89 eq) as an off-white powder after filtration.

^{1}H-NMR: (400 MHz, Chloroform-*d*) δ 3.64 (s, 808H, **C**H_2-**C**H_2-O), 2.69 (dt, J = 8.2, 6.4 Hz, 4H, O-CH_2-**C**H_2-SH), 1.59 (t, J = 8.2 Hz, 2H, -S**H**).

^{13}C-NMR: (101 MHz, Chloroform-*d*) δ 72.94, 70.70, 70.63 & 70.30 (**C**H_2-O), 24.33 (**C**H_2-SH).

*2.10. Synthesis of PEG-10k-OTs (**10**)*

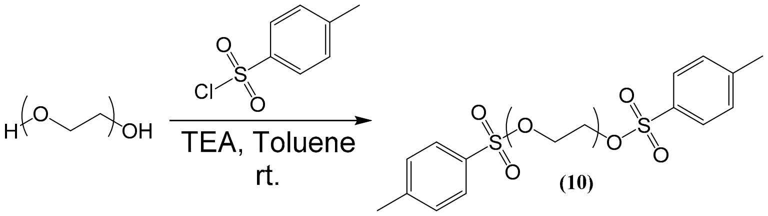

15.0 g (1.50 mmol, 1 eq) of 10 kDa PEG was dissolved in 75 mL of toluene and 4.2 mL of TEA (30.0 mmol, 20 eq) at 45°C. 4.3 g (22.5 mmol, 15 eq) of tosyl chloride was added, and the reaction was carried out for 14 hours at 45°C. The reaction mixture was then diluted with 300 mL of toluene in an extraction funnel and allowed to cool to room temperature before being washed with 4 x 100 mL of 10% $NaHSO_4$ in H_2O, 2 x 100 mL of deionized water and once with 100 mL of brine. The organic phase was dried with $MgSO_4$ filtered and rotary evaporated. The resulting oil was dissolved in dichloromethane and precipitated in ether twice, after which 12.2 g (1.22 mmol, 0.81 eq) of the product was obtained as a white powder.

^{1}H-NMR: (400 MHz, Chloroform-*d*) δ 7.79 (d, J = 8.1 Hz, 4H, C**H**=C(S)-C**H**), 7.34 (d, J = 8.1 Hz, 5H, C**H**=C(Me)-C**H**), 4.15 (t, J = 4.9 Hz, 5H, C**H**$_2$-O-S), 3.64 (s, 960H, C**H**$_2$-C**H**$_2$-O), 3.58 (s, 8H, C**H**$_2$-C**H**$_2$-O), 2.44 (s, 6H, C-C**H**$_3$).

^{13}C-NMR: (101 MHz, Chloroform-*d*) δ 144.52 (**C**-S(=O)$_2$-O), 132.83 (**C**-CH_3), 129.61 (**C**H=C(Me)-**C**H), 127.73 (**C**H=C(S)-**C**H), 70.49, 70.34, 69.05 & 68.42 (**C**H_2-**C**H_2-O), 21.43 (C-**C**H_3).

*2.11. Synthesis of PEG-10k-SAc (**11**)*

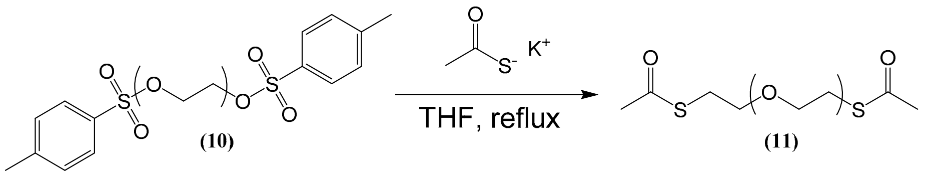

6.00 g (600 µmol, 1 eq) of tosylated 10 kDa PEG was dissolved in 24 mL of THF. 684 mg (6 mmol, 10 eq) of potassium thioacetate was added, and the mixture was refluxed for 14 hours. The resulting red/brown mixture was added to an extraction funnel along with 100 mL of deionized water, and this THF/water phase was treated with 3 x 100 mL of dichloromethane. The combined organic phases were then washed with 4 x 75 mL of 10% $NaHCO_3$ in water. The organic phase was dried with $MgSO_4$, rotary evaporated and the product was precipitated in ether. The obtained powder was dissolved in dichloromethane and precipitated again in ether to give 4.2 g (420 µmol, 0.70 eq) of product as a slightly brown powder after filtration.

^{1}H-NMR: (400 MHz, Chloroform-*d*) δ 3.64 (s, 1020H, C**H**$_2$-C**H**$_2$-O), 3.08 (t, J = 6.5 Hz, 4H, CH_2-C**H**$_2$-S), 2.33 (s, 6H, C(=O)-C**H**$_3$).

^{13}C-NMR: (101 MHz, Chloroform-*d*) δ 195.33 (S-**C**(=O)-CH_3), 70.50, 70.25 & 69.68 (**C**H_2-**C**H_2-O), 30.50 (CH_2-**C**H_2-S), 28.77 (S-C(=O)-**C**H_3).

*2.12. Synthesis of PEG-10k-SH (**12**)*

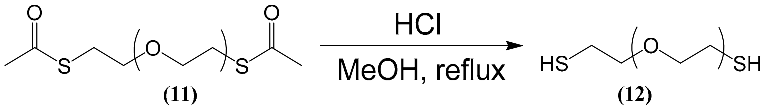

4.1 g (410 µmol, 1 eq) of 10 kDa thioacetate PEG was dissolved in 110 mL of methanol. 3 mL of concentrated hydrochloric acid was added, and the solution was refluxed for 3 hours. The product was precipitated in ether, dissolved in dicholoromethane and precipitated once more in ether, which gave 3.3 g (330 µmol, 0.80 eq) as an off-white powder after filtration.

^{1}H-NMR: (400 MHz, Chloroform-*d*) δ 3.64 (s, 1224H, C**H**$_2$-C**H**$_2$-O), 2.69 (dt, J = 8.2, 6.4 Hz, 4H, O-CH_2-C**H**$_2$-SH), 1.59 (t, J = 8.2 Hz, 2H, -S**H**).

^{13}C-NMR: (101 MHz Chloroform-*d*) δ 72.85 & 70.55 (**C**H_2-O), 24.24 (**C**H_2-SH).

*2.13. Synthesis of mPEG-5k-OTs (**13**)*

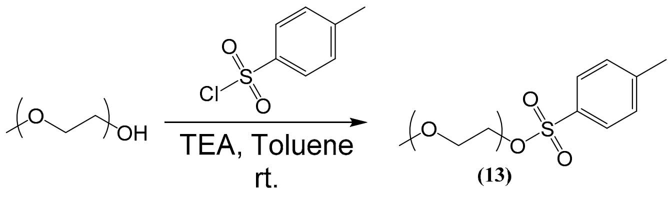

5.0 g (1 mmol, 1 eq) of 5 kDa monomethyl PEG was dissolved in 12.5 mL of toluene and 1.4 mL of TEA (10 mmol, 10 eq) at 45°C. 1.43 g (7.5 mmol, 7.5 eq) of tosyl chloride was added, and the reaction was carried out for 14 h at 45°C. The reaction mixture was then diluted with 300 mL of toluene and allowed to cool to room temperature before being added to an extraction funnel where it was washed with 4 x 75 mL of 10% $NaHSO_4$. The organic phase was dried with $MgSO_4$ filtered and rotary evaporated. The resulting oil was dissolved in dichloromethane and precipitated in ether twice. Filtration gave 4.1 g (790 µmol, 0.79 eq) of product as a white powder.

^{1}H-NMR: (400 MHz, Chloroform-*d*) δ 7.79 (d, J = 8.0 Hz, 3H, C**H**=C(S)-C**H**), 7.34 (d, J = 8.0 Hz, 3H, C**H**=C(Me)-C**H**), 4.24 – 4.08 (m, 3H, C**H**$_2$-O-S), 3.64 (s, 491H, C**H**$_2$-C**H**$_2$-O), 3.58 (s, 4H, O-C**H**$_3$), 3.54 (dd, J = 5.9, 3.6 Hz, 3H, CH_2-C**H**$_2$-O), 3.38 (d, J = 0.7 Hz, 3H, C-C**H**$_3$), 2.44 (s, 4H, C-C**H**$_3$).

^{13}C-NMR: (101 MHz, Chloroform-*d*) δ 144.56 (**C**-S(=O)$_2$-O), 132.89 (**C**-CH_3), 129.65 (**C**H=C(Me)-**C**H), 127.77 (**C**H=C(S)-**C**H**)**, 72.68, 71.75, 70.54, 70.39, 70.32, 69.08, 68.48, 68.09 & 63.49 (**C**H_2-**C**H_2-kkO), 58.83 (O-CH_3), 21.47 (C-**C**H_3).

*2.14. Synthesis of mPEG-5k-Sac (**14**)*

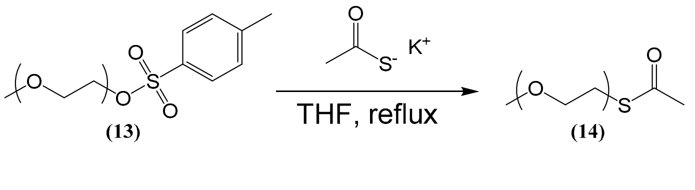

2.50 g (482 µmol, 1 eq) of tosylated 5 kDa monomethyl-PEG was dissolved in 5 mL of THF. 275 mg (2.41 mmol, 5 eq) of potassium thioacetate was added, and the mixture was refluxed for 14 hours. The resulting red/brown mixture was added to an extraction funnel along with 40 mL of deionized water, and this THF/water phase was treated with 3 x 50 mL of dichloromethane. The combined organic phases were then diluted to a total volume of 200 mL and washed with 4 x 50 mL of 10% $NaHCO_3$ in water. The organic phase was dried with $MgSO_4$, rotary evaporated and the product was precipitated in ether. The obtained powder was dissolved in dichloromethane and precipitated again in ether to give 2.1 g (408 µmol, 0.85 eq) of product as a slightly brown powder.

^{1}H-NMR: (400 MHz, Chloroform-d) δ 3.64 (s, 465H, **C**H_2-**C**H_2-O), 3.38 (s, 3H, O-**C**H_3), 3.09 (t, J = 6.5 Hz, 2H, CH_2-**C**H_2-S), 2.33 (s, 3H, C(=O)-**C**H_3).

^{13}C-NMR: (101 MHz, CDCl3) δ 71.97, 70.67, 70.60, 70.55, 70.35 & 69.78 (**C**H_2-**C**H_2-O), 59.06, 30.59 (CH_2-**C**H_2-S), 28.87 (S-C(=O)-**C**H_3).

*2.15. Synthesis of mPEG-5k-SH (**15**)*

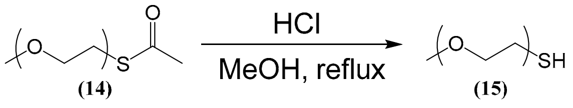

2.1 g (415 µmol) of 5 kDa thioacetate PEG was dissolved in 90 mL methanol. 2 mL of concentrated hydrochloric acid was added, and the solution was refluxed for 3 hours. The product was precipitated in ether, dissolved in dicholoromethane and precipitated once more in ether which gave the product as an off-white powder after filtration.

^{1}H-NMR: (400 MHz, Chloroform-d) δ 3.64 (s, 500H, **C**H_2-**C**H_2-O), 3.38 (s, 3H, O-**C**H_3), 2.88 (t, J = 6.5 Hz, 1H, O-**C**H_2-CH_2-SH), 2.70 (q, J = 6.9 Hz, 1H, O-CH_2-**C**H_2-SH), 1.59 (t, J = 8.1 Hz, 1H, -S**H**).

^{13}C-NMR: (101 MHz, Chloroform-d) δ 70.37 (**C**H_2-O).

*2.16. Synthesis of carboxylic functional disperse red 13 (**16**)*

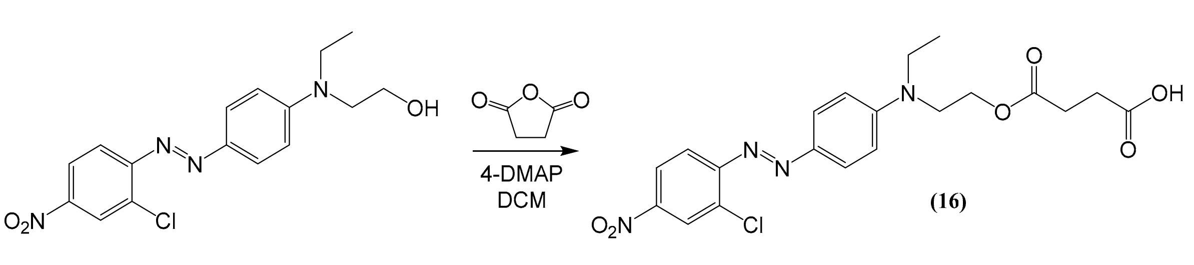

1.00 g (2.87 mmol, 1 eq) of disperse red 13 was dissolved in 2 mL of dichloromethane and 1 mL of pyridine. 70 mg (574 µmol, 0.2 eq) of 4-DMAP was added followed by 340 mg (3.40 mmol, 1.2 eq) of succinic anhydride. The reaction was allowed to proceed at room temperature for 3 hours. Excess succinic anhydride was converted into succinic acid by the addition of 1 mL of deionized water. This quenching was carried out for 19 hours at room temperature. The full conversion of excess succinic anhydride into succinic acid was confirmed by ^{13}C-NMR spectroscopy. The reaction mixture was diluted with 75 mL of dichloromethane, and the organic phase was treated four times with 15 mL of 10% $NaHSO_4$ in water followed by 4 x 15 mL of deionized water. The organic phase was dried with

$MgSO_4$. and rotary evaporated to give 1.15 g (2.44 mmol, 0.85 eq) of carboxylic functional disperse red 13 as a red solid.

^{1}H-NMR: (400 MHz, Chloroform-*d*) δ 8.38 (d, J = 2.4 Hz, 1H, C(NO_2)-C**H**=C(Cl)), 8.15 (dd, J = 8.9, 2.4 Hz, 1H, C(NO2)=C**H**-CH), 7.99 – 7.88 (m, 2H, C(N=N)-C**H**-CH-CN(Et)(EtOH)), 7.77 (d, J = 8.9 Hz, 1H, CH-C**H**=C(N=N)), 6.83 – 6.77 (m, 2H, C(N=N)-CH-C**H**-CN(Et)(EtO)), 4.33 (t, J = 6.2 Hz, 2H, N-CH_2-C**H₂**), 3.69 (t, J = 6.3 Hz, 2H, N-CH_2-C**H₂**), 3.53 (q, J = 7.1 Hz, 2H, C**H₂**-CH_3), 2.75 – 2.53 (m, 4H, C**H₂**-C**H₂**-COOH), 1.26 (t, J = 7.1 Hz, 3H, CH_2-C**H₃**).

^{13}C-NMR: (101 MHz, Chloroform-*d*) δ 177.32 (**C**OOH), 172.16 (COO), 153.20 (C(Cl)-**C**-N=N), 151.77 (**C**-N(Et)(EtO)), 147.38 (**C**-NO_2), 144.64 (N=N-**C**-CH), 134.16 (**C**-Cl), 127.09 (C(Cl)-C=**C**H), 126.16 (**C**H-CH-N(Et)(EtO)), 122.75 (C(NO_2)-**C**H-C(Cl)), 118.19 (C(NO_2)=**C**H-CH), 111.71 (**C**H-C-N(Et)(EtO)), 61.76 (N-CH_2-**C**H_2), 48.92 (N-**C**H_2-CH_2), 45.93 (N-**C**H_2-CH_3), 28.84 (**C**H_2-CH_2-(COOH)), 28.75 (**C**H_2-CH_2-(COOH)), 12.41 (N-CH_2-**C**H_3).

*2.17. Synthesis of NHS-activated carboxylic functional disperse red 13 (**17**)*

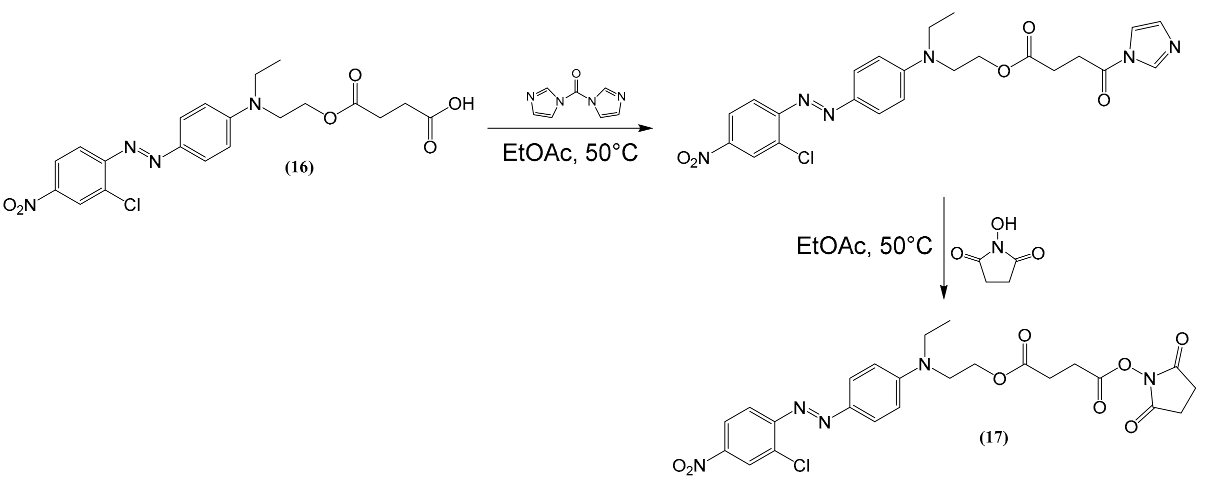

1.15 g (2.44 mmol, 1 eq) of carboxylic functional disperse red 13 was dissolved in 3 mL of dichloromethane. 395 mg (2.44 mmol, 1 eq) of CDI was added. After 1 hour, complete imidazole activation was confirmed by ^{1}H-NMR spectroscopy upon which 421 mg (3.66 mmol, 1.5 eq) of NHS was added. The reaction was carried out at room temperature for 14 hours. Complete reaction was confirmed with ^{1}H-NMR spectroscopy, upon which the reaction mixture was diluted with 75 mL of dichloromethane. The organic phase was treated with 4 x 15 mL of 10% $NaHSO_4$ and 15 mL of deionized water. The organic phase was dried with $MgSO_4$ and rotary evaporated to give 1.23 g (2.17 mmol, 0.89 eq) of product as a dark red solid.

^{1}H-NMR: (400 MHz, Chloroform-*d*) δ 8.39 (d, J = 2.4 Hz, 1H, C(NO_2)-C**H**=C(Cl))), 8.16 (dd, J = 8.9, 2.4 Hz, 1H, C(NO2)=C**H**-CH), 8.04 – 7.91 (m, 2H, C(N=N)-C**H**-CH-CN(Et)(EtOH))), 7.78 (d, J = 8.9 Hz, 1H, CH-C**H**=C(N=N))), 6.82 (dd, J = 9.6, 2.7 Hz, 2H, C(N=N)-CH-C**H**-CN(Et)(EtO))), 4.36 (t, J = 6.4 Hz, 2H, N-CH_2-C**H₂**), 3.72 (t, J = 6.3 Hz, 2H, N-C**H₂**-CH_2), 3.54 (q, J = 7.1 Hz, 2H, **C**H_2-CH_3), 2.94 (dd, J = 7.4, 6.2 Hz, 2H, CH_2-C**H₂**-COONHS), 2.84 (s, 4H, C**H₂**-C**H₂** (NHS)), 2.76 (dd, J = 7.5, 6.2 Hz, 2H, C**H₂**-CH_2-COONHS)), 1.26 (td, J = 7.1, 1.5 Hz, 4H, CH_2-C**H₃**).

^{13}C-NMR: (101 MHz, Chloroform-*d*) δ 170.91 (**C**=O)-CH_2-CH_2-COONHS), 168.96 (**C**=O (NHS)), 167.77 (C=O)-CH_2-CH_2-**C**OONHS), 153.24 (C(Cl)-**C**-N=N), 151.83 (**C**-N(Et)(EtO)), 147.37 (**C**-NO_2), 144.63

(N=N-**C**-CH), 134.15 (**C**-Cl), 127.10 (C(Cl)-C=**C**H), 126.16 (**C**H-CH-N(Et)(EtO)), 122.76 (C(NO$_2$)-**C**H-C(Cl)), 118.20 (C(NO$_2$)=C**H**-CH), 111.72 (C**H**-C-N(Et)(EtO)), 61.98 (N-CH$_2$-**C**H$_2$), 48.72 (N-**C**H$_2$-CH$_2$), 45.85 (N-**C**H$_2$-CH$_3$), 28.87 (**C**H$_2$-CH$_2$-(COONHS)), 26.44 (**C**H$_2$-CH$_2$-(COOH)), 25.72 (**C**H2-**C**H2 (NHS)), 12.43 (CH$_2$-**C**H$_3$).

*2.18. Synthesis of generation two acetonide protected AB$_2$C dendrimer (TMP-G2-(acetonide)$_6$(ene)$_6$) (**18**)*

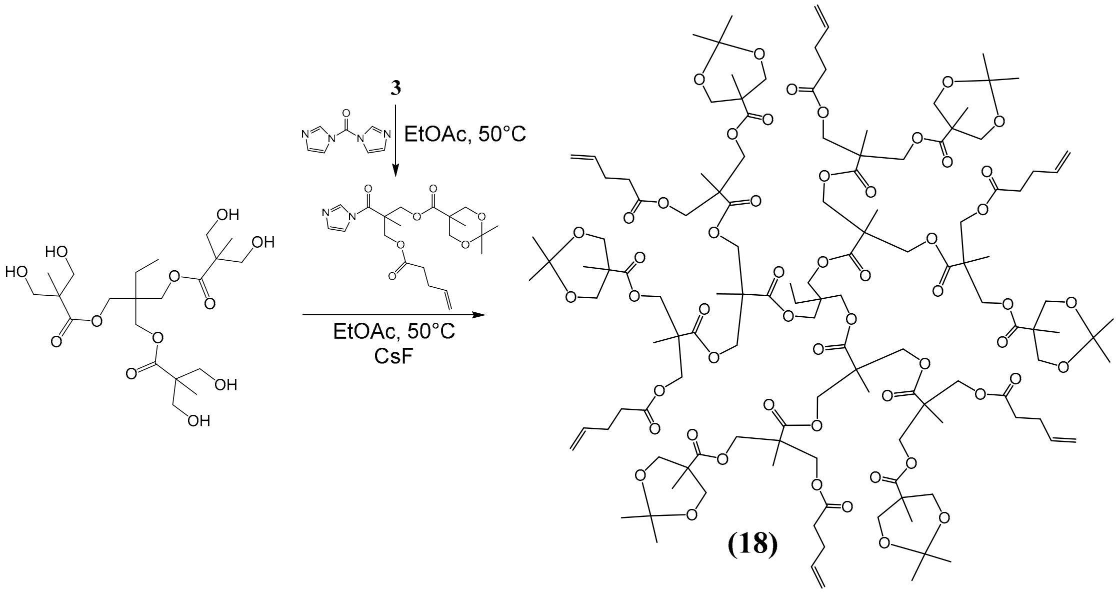

466 mg (1.30 mmol, 9 eq) of monomer **3** was dissolved in 1.5 mL of ethyl acetate and heated to 50°C. 211 mg (1.30 mmol, 9 eq) of CDI was added. The imidazole activation was carried out for 1 hour and completion was confirmed by ^{1}H-NMR spectroscopy. 70 mg (145 µmol, 9 eq) of generation 1 bis-MPA dendrimer[1] was added together with 26 mg (174 µmol, 1.2 eq) of cesium fluoride. The reaction was carried out at 50°C under nitrogen for 4 hours and completion was confirmed with MALDI-TOF-MS. 0.5 mL of deionized water was added to quench the excess imidazole-activated monomer. Quenching was carried out for 14 hours at room temperature. The organic phase was diluted with 75 mL of ethyl acetate and washed with 5 x 20 mL of 10% sodium bisulfate in water, 5 x 20 mL of 10% sodium bicarbonate in water and 20 mL of brine. The organic phase was dried with $MgSO_4$ and rotary evaporated to give 310 mg (119 mmol, 0.82 eq) of pure product as a clear oil.

^{1}H-NMR: (400 MHz, Chloroform-*d*) δ 5.87 – 5.70 (m, 6H, C**H**=CH$_2$), 5.09 – 4.93 (m, 12H, CH=C**H$_2$**), 4.35 – 4.04 (m, 54H, C-C**H$_2$**-O), 3.61 (d, J = 11.7 Hz, 14H, C-C**H$_2$**-O), 2.38 (dt, J = 27.1, 7.3 Hz, 28H, C**H$_2$**-C**H$_2$**), 1.54 (d, J = 11.8 Hz, 4H, C**H$_2$**-CH$_3$), 1.38 (d, J = 25.0 Hz, 36H, C-(C**H$_3$**)$_2$), 1.27 (s, 9H, C-C**H$_3$**), 1.25 (s, 19H, C-C**H$_3$**), 1.14 (s, 18H,C-C**H$_3$**), 0.95 (t, J = 7.5 Hz, 3H, CH$_2$-C**H$_3$**).

^{13}C-NMR: (101 MHz, Chloroform-*d*) δ 173.64, 172.37, 171.98 & 171.71 (**C**OO), 136.57, (**C**H=CH$_2$), 115.76 (CH=**C**H$_2$), 98.20 (**C**-(CH3)$_2$), 66.08, 66.03, 65.31, 65.02 & 64.22 (C-**C**H$_2$-O), 46.92, 46.71 & 42.16 (**C**-CH$_3$), 41.59 (**C**-CH$_2$-CH$_3$), 33.29 (**C**H$_2$-CH$_2$-CH), 28.78 (CH$_2$-**C**H$_2$-CH), 25.29 (C-(**C**H$_3$)$_2$), 22.18 (C-(**C**H$_3$)$_2$), 18.60, 17.88 & 17.67 (C-**C**H$_3$), 7.64 (CH$_2$-**C**H$_3$).

MALDI: (Mass+Na$^+$)$_{calc}$: 2630 Da (Mass+Na$^+$)$_{found}$: 2631 Da

*2.19. Synthesis of hydroxyl functional AB_2C-dendrimer (TMP-G2-$(OH)_{12}(ene)_6$) (**19**)*

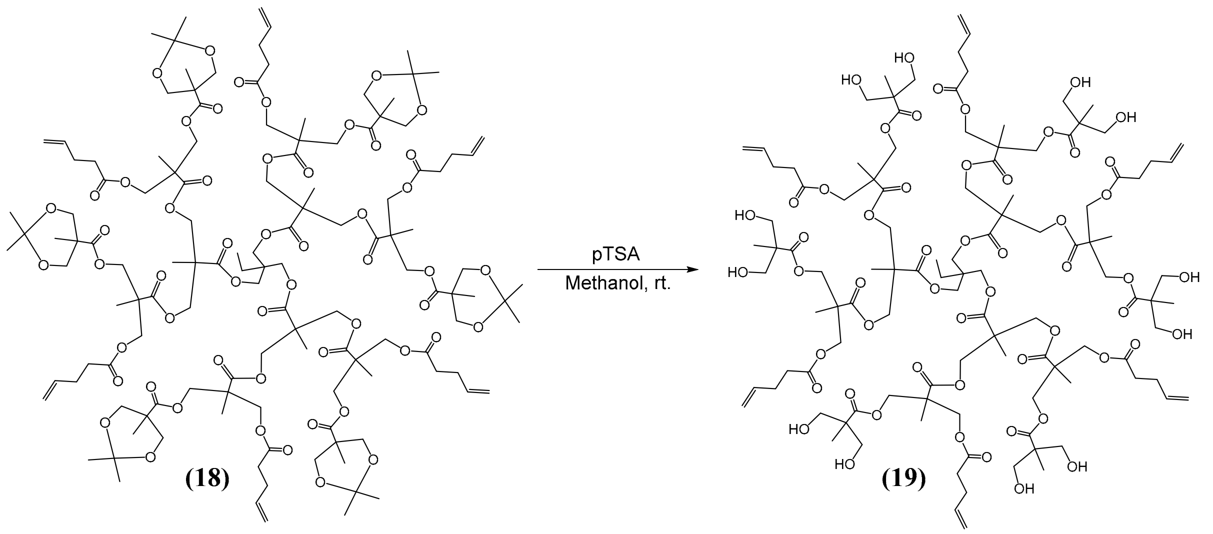

310 mg (119 mmol, 1 eq) of **4** was dissolved in 90 mL of methanol. 31 mg (10 wt.%) of pTSA was added. The reaction mixture was rotary evaporated at 30°C until around 75% of the methanol had been removed. The reaction flask was refilled with methanol and the rotary evaporation was repeated three times. Completion was confirmed with MALDI-TOF MS and the pTSA was removed by filtering the reaction mixture through a column of Amberlyst A21 free base that had previously been cleaned by being stirred for 2 hours sequentially in dichloromethane, ethyl acetate and methanol. After rotary evaporation of the methanol, 258 mg (109 mmol, 0.92 eq) of pure product was obtained as a clear viscous oil.

^{1}H-NMR (400 MHz, Methanol-*d*4) δ 5.84 (ddt, J = 16.8, 11.4, 6.3 Hz, 6H, C**H**=CH_2), 5.11 – 4.94 (m, 13H, CH=C**H_2**), 4.40 – 4.15 (m, 42H, C-C**H_2**-O), 3.65 (q, J = 10.9 Hz, 24H, C-C**H_2**-O), 2.41 (dt, J = 39.3, 7.2 Hz, 24H, C**H_2**-C**H_2**), 1.62 (q, J = 7.4 Hz, 2H, C**H_2**-CH_3), 1.34 (s, 9H, C-C**H_3**), 1.29 (s, 19H, C-C**H_3**), 1.15 (s, 18H, C-C**H_3**), 1.00 (t, J = 7.4 Hz, 3H, CH_2-C**H_3**).

^{13}C-NMR: (101 MHz, Methanol-*d*4) δ 175.88, 174.05, 173.65, 173.40 (**C**OO), 137.99 (**C**H=CH_2), 116.10 (CH=**C**H_2), 66.81, 66.55, 66.17, 65.78 & 65.47 (C-**C**H_2-O), 51.71, 48.19 & 47.87 (**C**-CH_3) & 42.79 (**C**-CH_2-CH_3), 34.26 (**C**H_2-CH_2-CH), 29.87 (CH_2-**C**H_2-CH), 18.31, 18.19 & 17.41 (C-**C**H_3), 8.17 (CH_2-**C**H_3).

MALDI: $(Mass+Na^+)_{calc}$: 2390 Da $(Mass+Na^+)_{found}$: 2392 Da

*2.20. Synthesis of boc-protected amino functional AB_2C-dendrimer (TMP-G2-$(NHBoc)_{12}(ene)_6$) (**20**)*

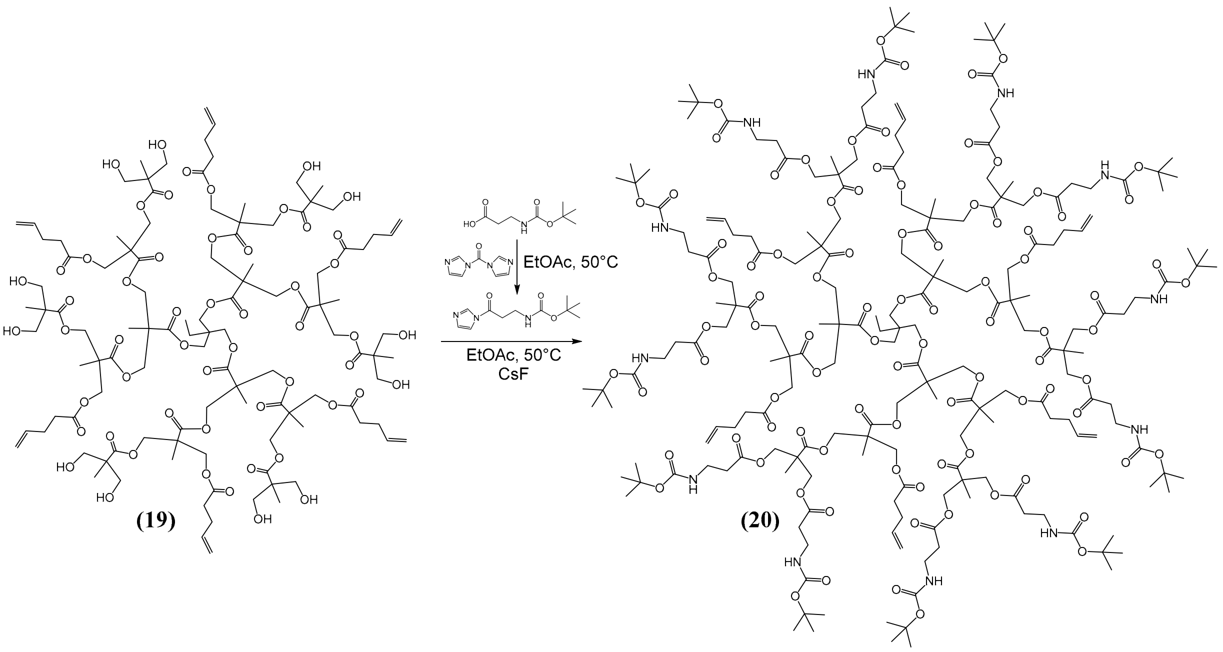

(19) **(20)**

514 mg (2.72 mmol, 25 eq) of boc-protected beta-alanine was dissolved in 3 mL of ethyl acetate and heated to 50°C before 440 mg (2.72 mmol, 25 eq) of CDI was added. The imidazole activation was carried out for 1 hour and completion was confirmed with ^{1}H-NMR spectroscopy. The solution was added to 258 mg (109 µmol, 1 eq) of dendrimer **5** and 40 g (262 µmol, 2.4 eq) of cesium fluoride was added. The reaction was carried out for 14 hours at 50°C and completion was confirmed with MALDI-TOF MS. 1 mL of deionized water was added to quench the remaining imidazole-activated beta-alanine. The quenching was carried out for 6 hours at room temperature. The organic phase was diluted with 75 mL of ethyl acetate and washed with 5 x 20 mL of 10% sodium bisulfate in water, 5 x 20 mL of 10% sodium bicarbonate in water and 20 mL of brine. The organic phase was dried with $MgSO_4$ and rotary evaporated to give 403 mg (91 µmol, 0.83 eq) of pure product as a clear oil.

^{1}H-NMR: (400 MHz, Chloroform-*d*) δ 5.84 – 5.69 (m, 6H, C**H**=CH_2), 5.25 – 5.15 (m, 10H, N**H**), 5.07 – 4.93 (m, 12H, CH=C**H₂**), 4.32 – 4.04 (m, 66H, C-C**H₂**-O), 3.34 (q, J = 6.0 Hz, 24H, C**H₂**-CH_2-NHBoc), 2.51 (t, J = 6.0 Hz, 25H, CH_2-C**H₂**-NHBoc), 2.36 (dt, J = 30.1, 7.3 Hz, 24H, C**H₂**-C**H₂**-CH), 1.41 (s, 111H, C-(C**H₃**)₃), 1.26 (s, 8H, C-C**H₃**), 1.21 (s, 38H, C-C**H₃**), 0.93 (t, J = 7.5 Hz, 3H, CH_2-C**H₃**).

^{13}C-NMR: (101 MHz, Chloroform-*d*) δ 172.35, 172.12, 171.91, 171.81 & 171.68 (COO/CON), 155.90 (O-(**C**=O)-NH), 136.48 (**C**H=CH_2), 115.83 (CH=**C**H₂), 79.42 (**C**-$(CH_3)_3$), 65.46, 65.30, 64.89, 64.83 & 64.20 (C-**C**H₂-O), 46.87, 46.58, 46.53 (**C**-CH_3) & 41.59 (**C**-CH_2-CH_3), 36.19 (CH_2-C**H₂**-NH), 34.54 (C**H₂**-CH_2-CH), 33.22 (C**H₂**-CH_2-NH), 28.72, 28.49, 17.95, 17.80, 17.65 (C-**C**H₃), 7.62 (CH_2-C**H₃**).

MALDI: $(Mass+Na^+)_{calc}$: 4443 Da $(Mass+Na^+)_{found}$: 4444 Da

*2.21. Synthesis of trifluoroacetate salt of amino functional AB_2C-dendrimer (TMP-G2-($NH_3^+TFA^-$)$_{12}$(ene)$_6$) (**21**)*

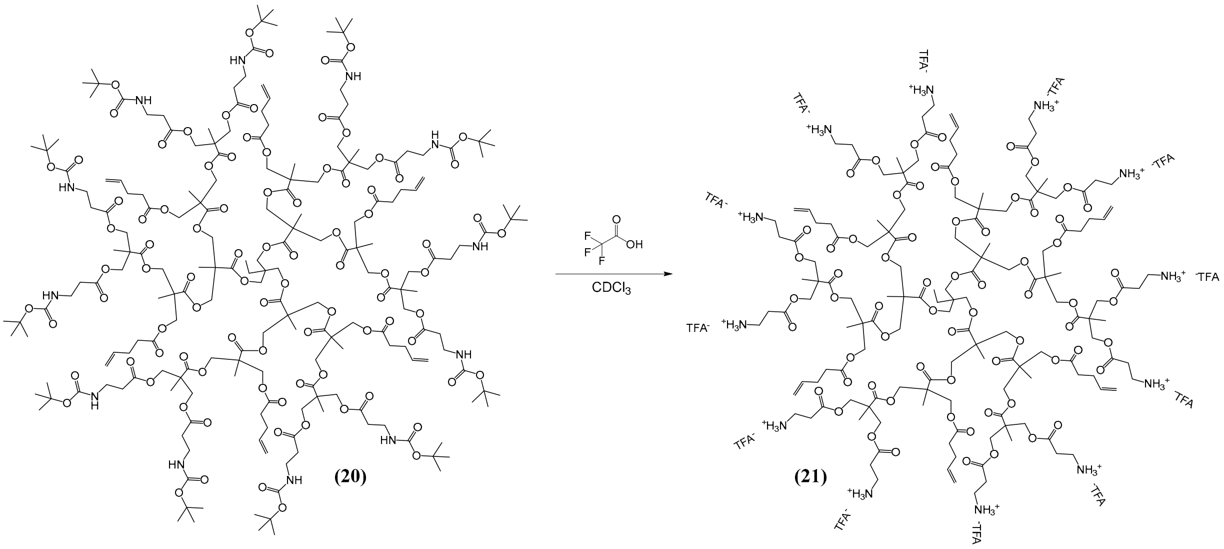

403 mg (91 µmol, 1 eq) of dendrimer **6** was dissolved in 1 mL of a 1:1 mixture of trifluoroacetic acid and chloroform. The reaction was carried out for 1 hour at room temperature and full conversion was confirmed by MALDI-TOF MS analysis, where the dendrimer was detected as a freebase since it was operated in positive mode. The TFA and chloroform were removed under reduced pressure. The dendrimer was dissolved in deionized water and treated once with ether before freeze-drying to ensure removal of solvent traces before cell and bacterial tests were carried out. 374 mg (81 µmol, 0.89 eq) was obtained as a sticky solid.

^{1}H-NMR: (400 MHz, Methanol-*d*4) δ 5.84 (ddt, J = 16.6, 10.3, 6.3 Hz, 5H, C**H**=CH_2), 5.13 – 4.94 (m, 13H, C**H**=CH_2), 4.42 – 4.09 (m, 64H, C-C**H_2**-O), 3.23 (t, J = 6.7 Hz, 23H, CH_2-C**H_2**-NH_3^+), 2.80 (t, J = 6.7 Hz, 23H, C**H_2**-CH_2-NH_3^+), 2.41 (dt, J = 41.2, 7.3 Hz, 24H, C**H_2**-C**H_2**-CH), 1.62 (s, 3H, C**H_2**-CH_3), 1.40 – 1.24 (m, 44H, C-C**H_3**), 1.00 (t, J = 7.3 Hz, 4H, CH_2-C**H_3**).

^{13}C-NMR: (101 MHz, Methanol-*d*4) δ 174.05, 173.45 & 171.76 (**C**OO/**C**ON), 163.16, 162.81 & 162.46 (C=O)-CF_3, 137.98 (**C**H=CH_2), 119.46 ((C=O)-**C**F_3), 116.56 & 116.22 (CH-**C**H_2), 66.82, 66.26 (C-**C**H_2-O), 48.17, 47.85 & 47.67 (**C**-CH_3), 36.29 (CH_2-C**H_2**-NH_3^+), 34.24 (C**H_2**-CH_2-NH_3^+), 32.15 (C**H_2**-CH_2-CH), 29.85 (CH_2-C**H_2**-CH), 18.25 & 18.15 (C-**C**H_3).

*MALDI: (Mass+Na^+)$_{calc}$: 3242 Da (Mass+Na^+)$_{found}$: 3243 Da

*The MALDI was run in positive mode, and the dendrimer was detected in the completely deprotonated form.

3. Model thiol-ene reaction between the second-generation ammonium functional dendrimer and monomethylated 5 kDa thiolated PEG

The second-generation dendrimer and mPEG-5k-SH were reacted under the same conditions as used with the 2, 5 and 10 kDa dithiol functional PEGs. A 1:6 molar ratio of dendrimer to mPEG-5k-SH was used for the reaction. 0.5 wt.% LAP was used as the photo-initiator and UV curing with λ = 356 nm was applied for the TEC reaction. After the reaction, the product was purified by freeze-drying. The progress of the thiol-ene reaction was investigated by ^{1}H NMR spectroscopy by dissolving the reaction mixture in deuterated methanol at irradiation times of t = 0, 15 and 30 minutes. A comparison of these spectra with the ^{1}H NMR spectra of mPEG-5k-SH, dendrimer and the crosslinked product after purification by freeze-drying are shown in Figures S2-4.

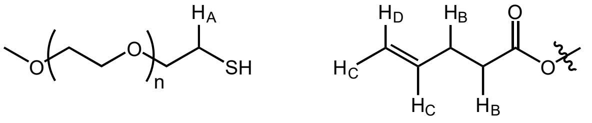

Figure S1. Positions of protons A-D in Figures S3 and S4.

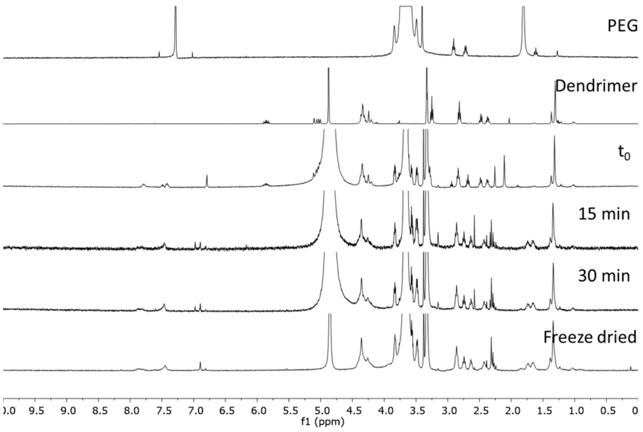

Figure S2. ^{1}H NMR spectra in deuterated methanol showing the thiol-ene reaction between mPEG-5k-SH and the second generation dendrimer at irradiation times of t = 0, 15 and 30 minutes, and the spectrum of the product after purification by freeze-drying.

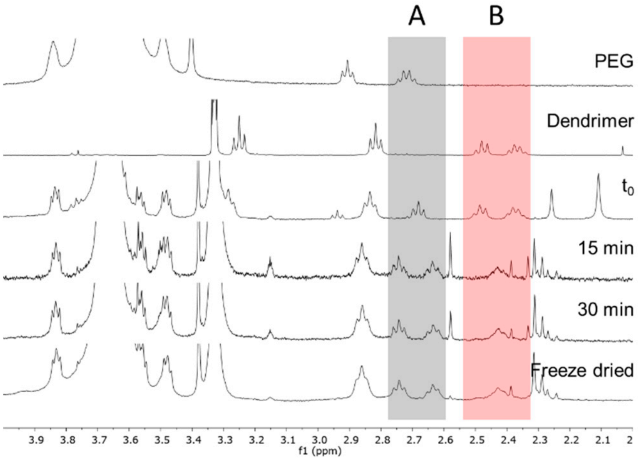

Figure S3. ^{1}H NMR spectra in deuterated methanol, focusing on the 2-4 ppm spectral window, showing the shift of protons A (CH_2 adjacent to the thiol) and B during the thiol-ene reaction between mPEG-5k-SH and the second generation dendrimer.

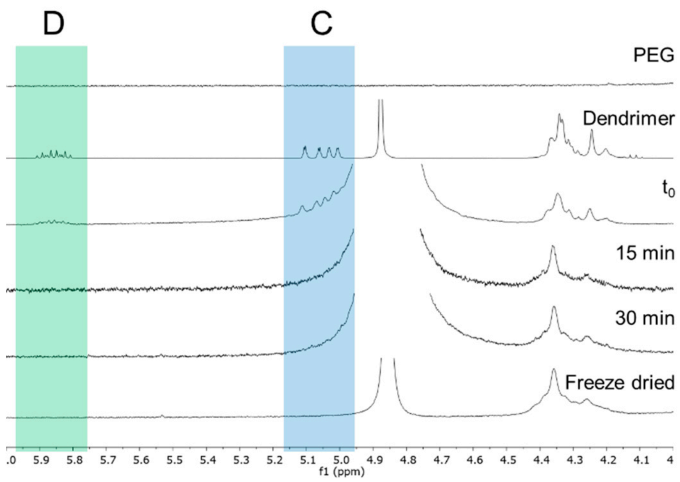

Figure S4. ^{1}H NMR spectra in deuterated methanol, focusing on the 4-6 ppm spectral window, showing the shift of protons C and D during the thiol-ene reaction between mPEG-5k-SH and the second generation dendrimer.

4. NMR and MALDI spectra of synthesized compounds

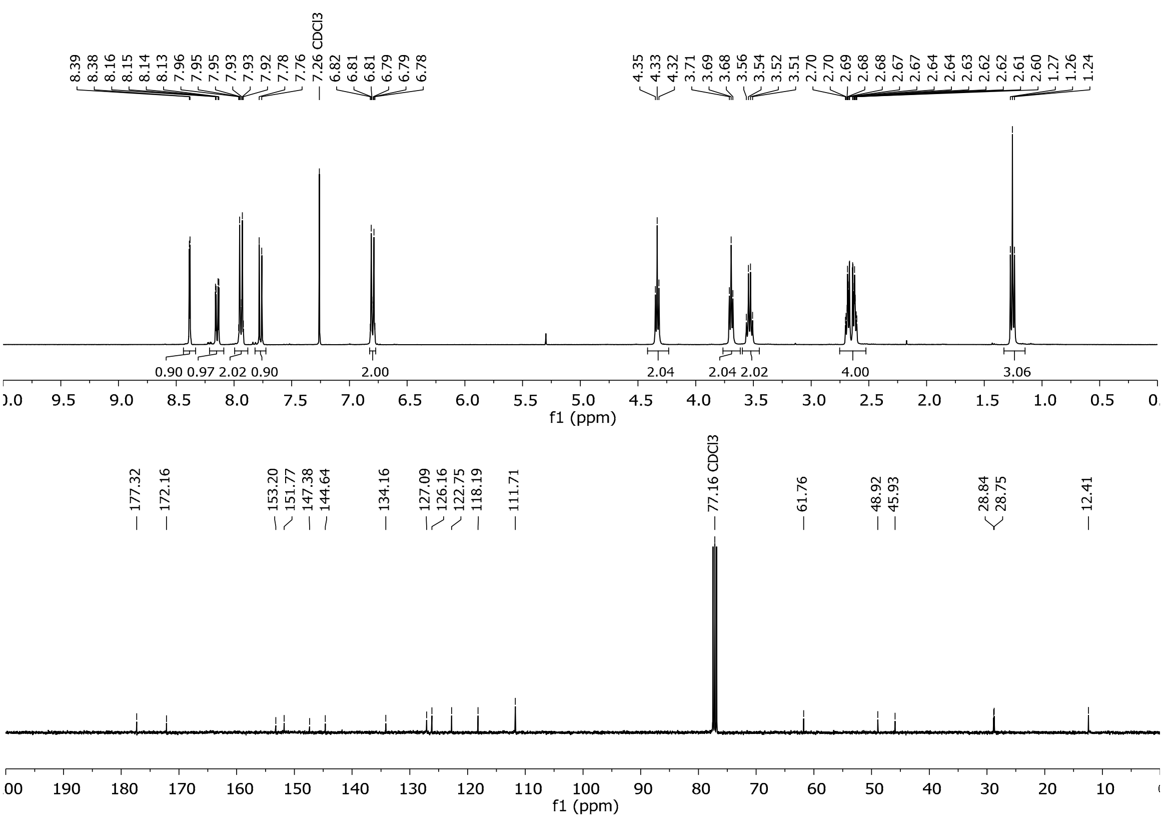

Figure S5. ^{1}H and ^{13}C NMR spectra of carboxylic acid functional disperse red 13 in deuterated chloroform.

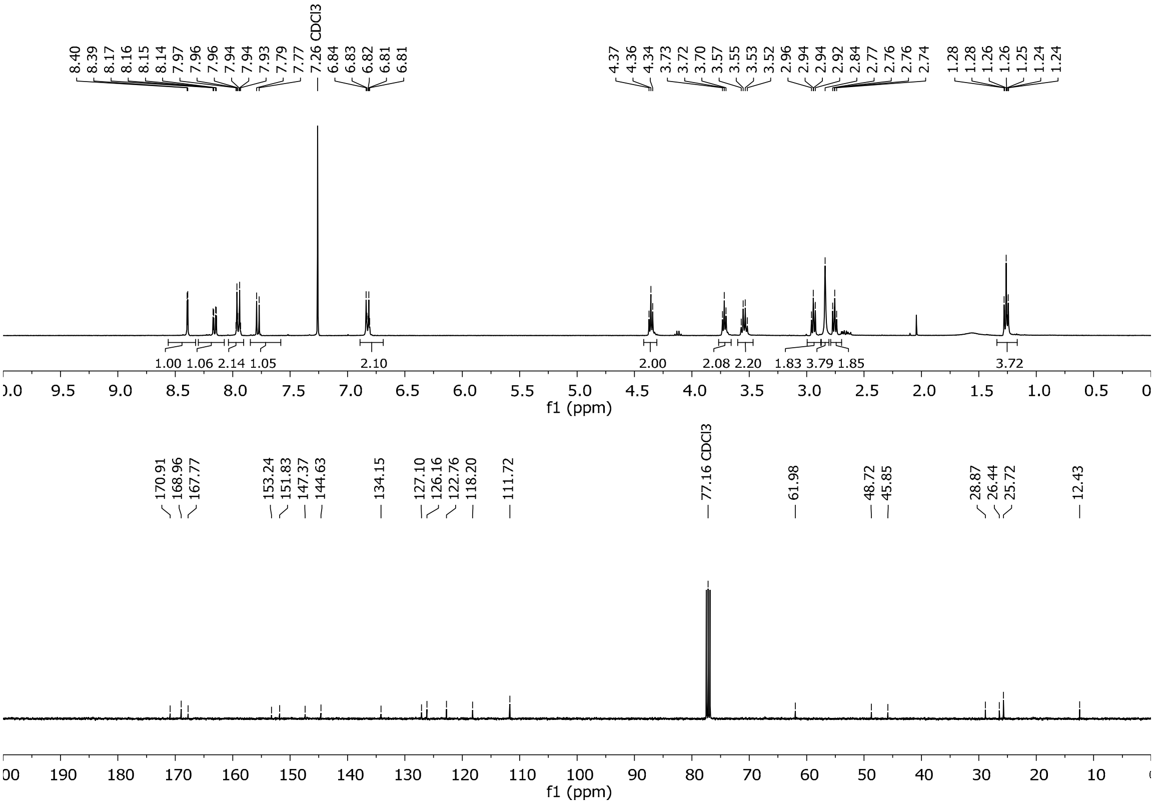

Figure S6. ^{1}H and ^{13}C NMR spectra of NHS-activated carboxylic acid functional disperse red 13 in deuterated chloroform.

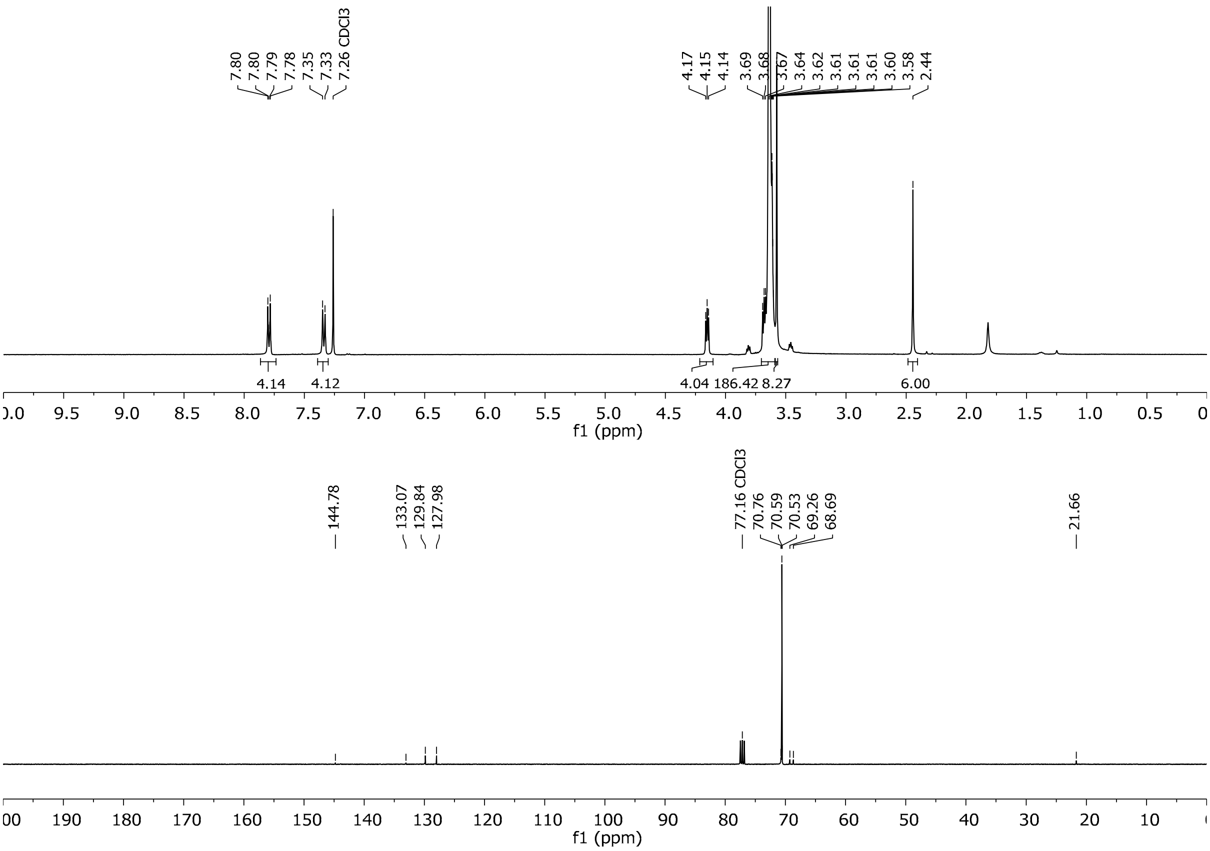

Figure S7. ^{1}H and ^{13}C NMR spectra of PEG2k-OTs in deuterated chloroform.

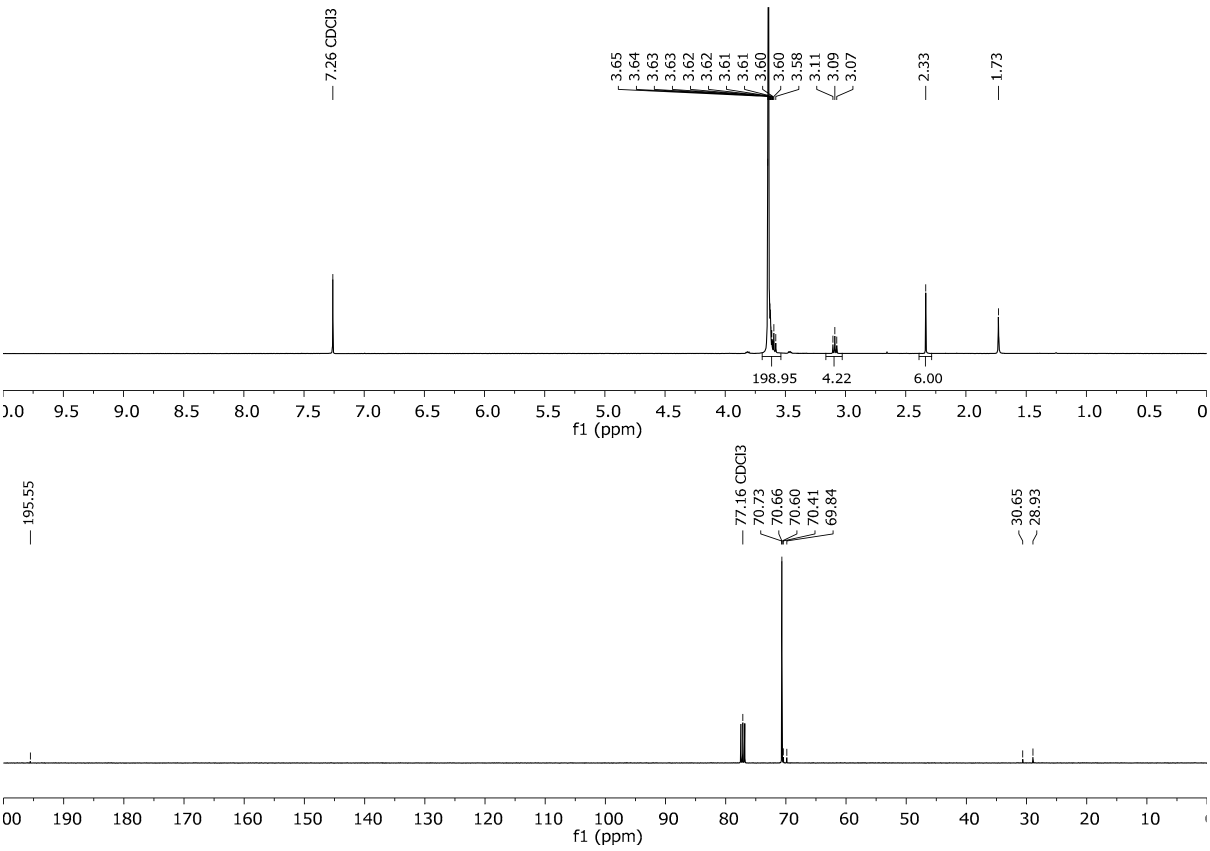

Figure S8. ^{1}H and ^{13}C NMR spectra of PEG2k-SAc in deuterated chloroform.

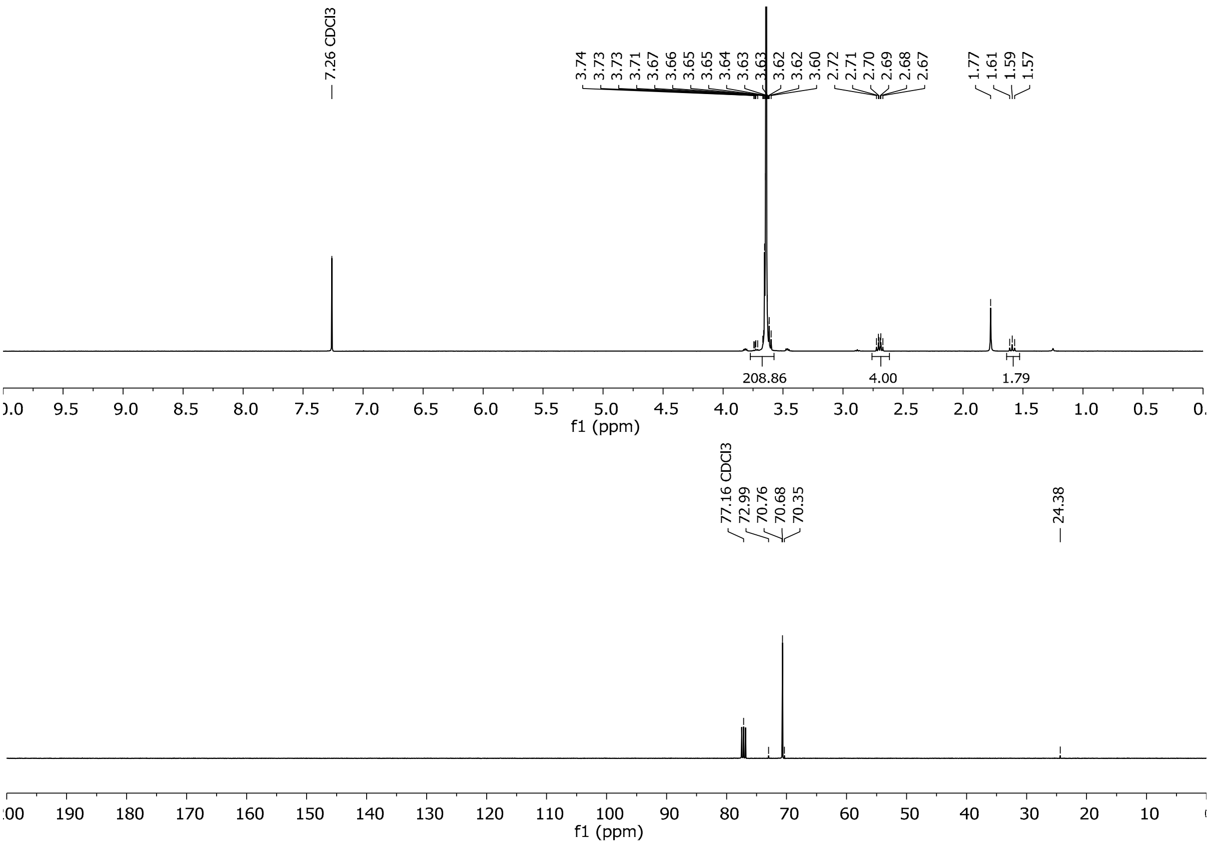

Figure S9. ^{1}H and ^{13}C NMR spectra of PEG2k-SH in deuterated chloroform.

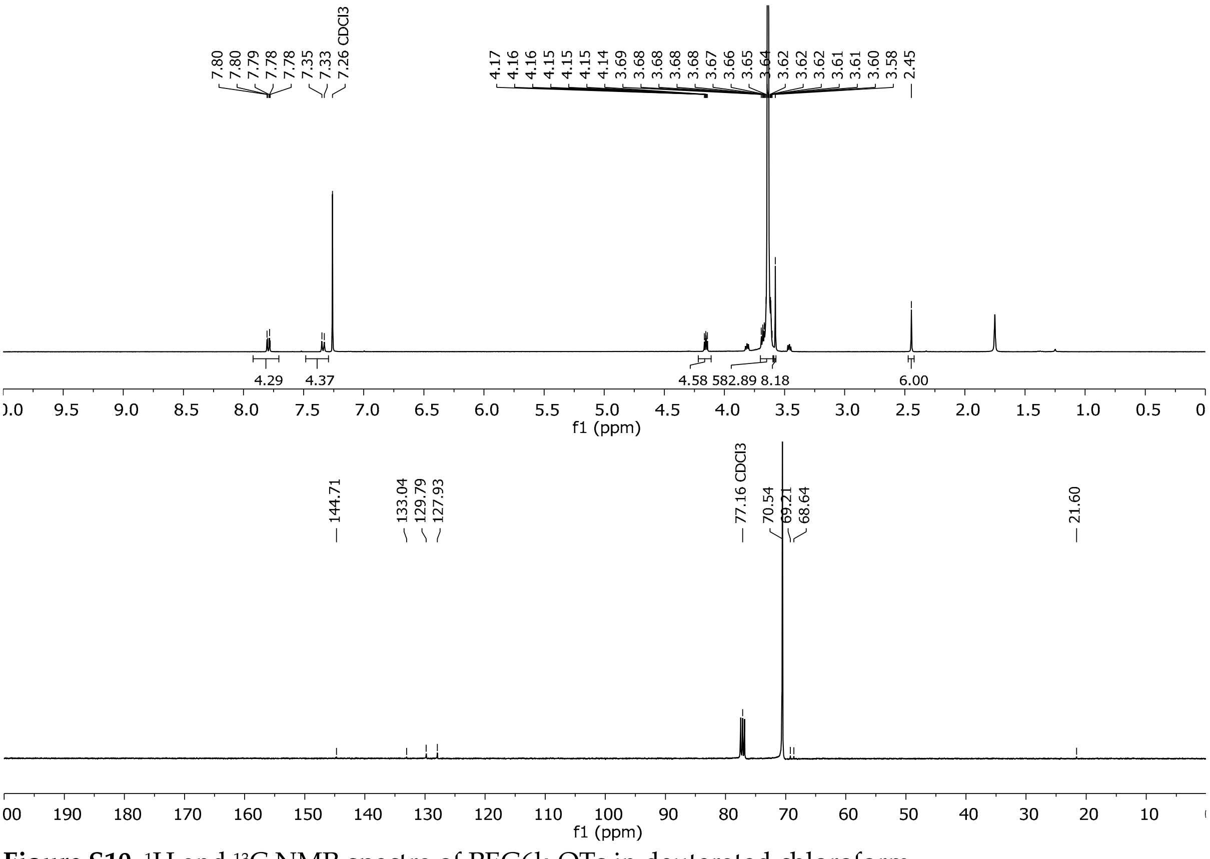

Figure S10. ^{1}H and ^{13}C NMR spectra of PEG6k-OTs in deuterated chloroform.

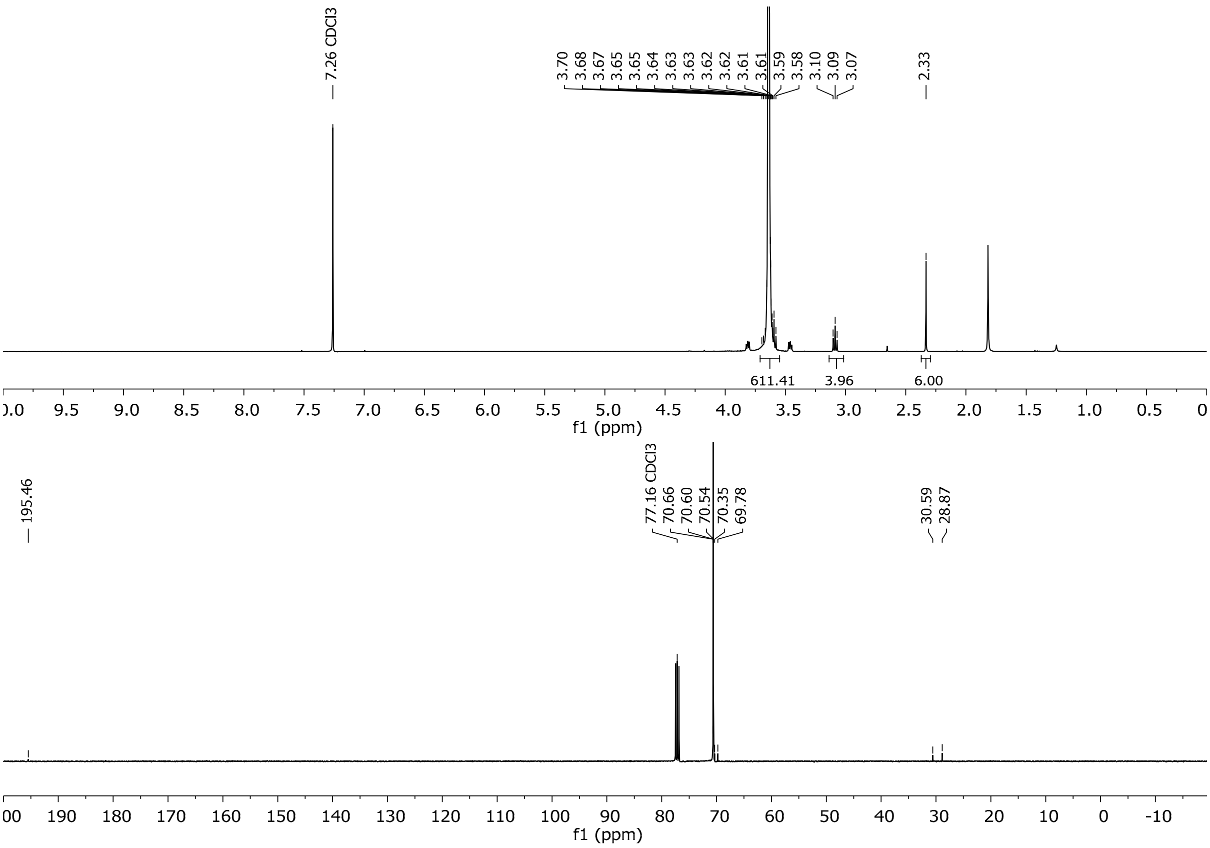

Figure S11. ^{1}H and ^{13}C NMR spectra of PEG6k-SAc in deuterated chloroform.

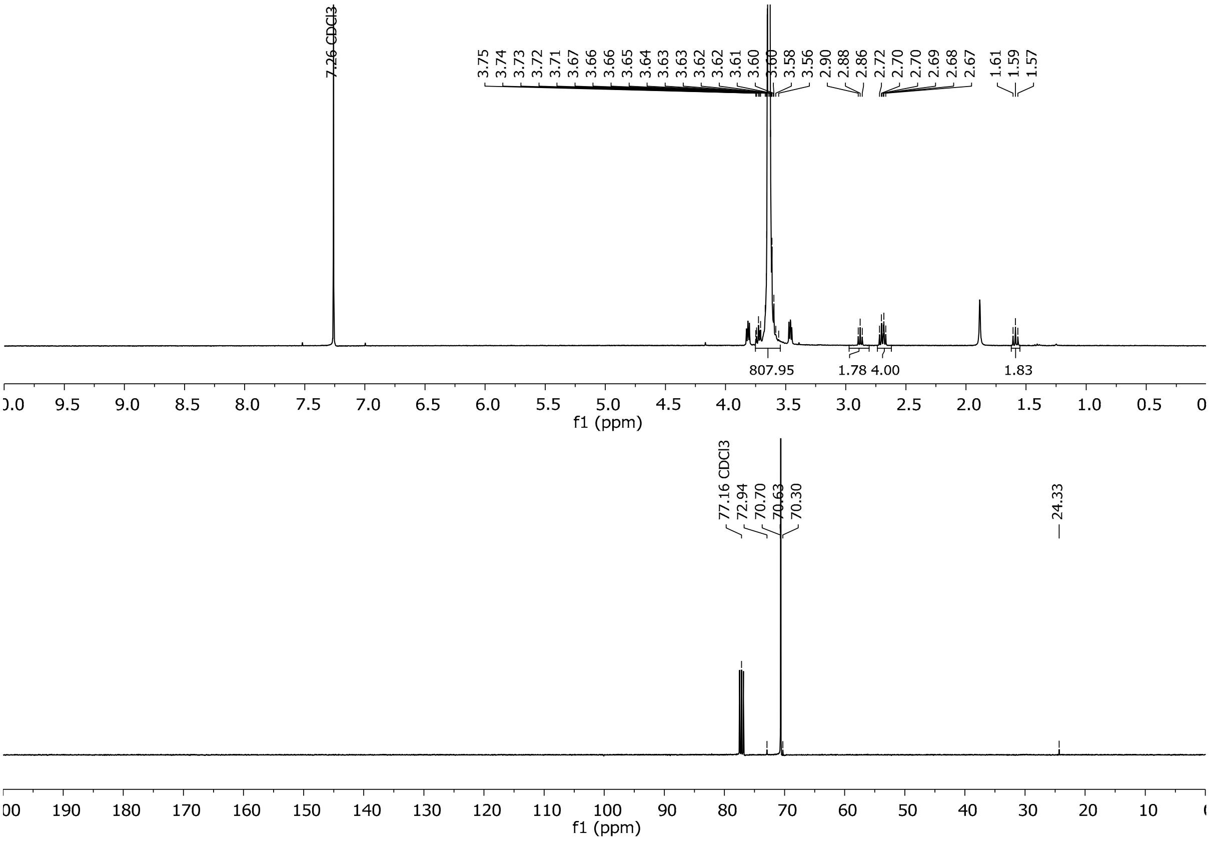

Figure S12. ^{1}H and ^{13}C NMR spectra of PEG6k-SH in deuterated chloroform.

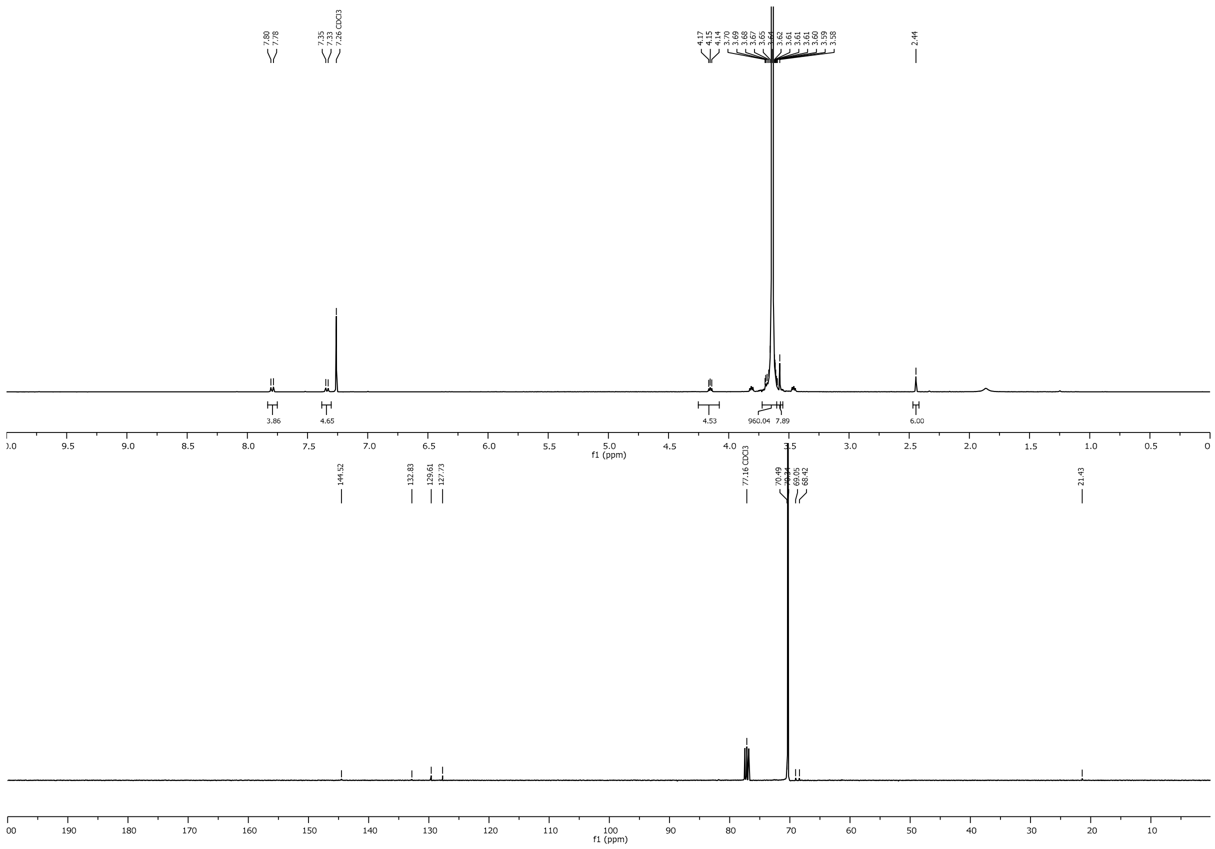

Figure S13. ^{1}H and ^{13}C NMR spectra of PEG10k-OTs in deuterated chloroform.

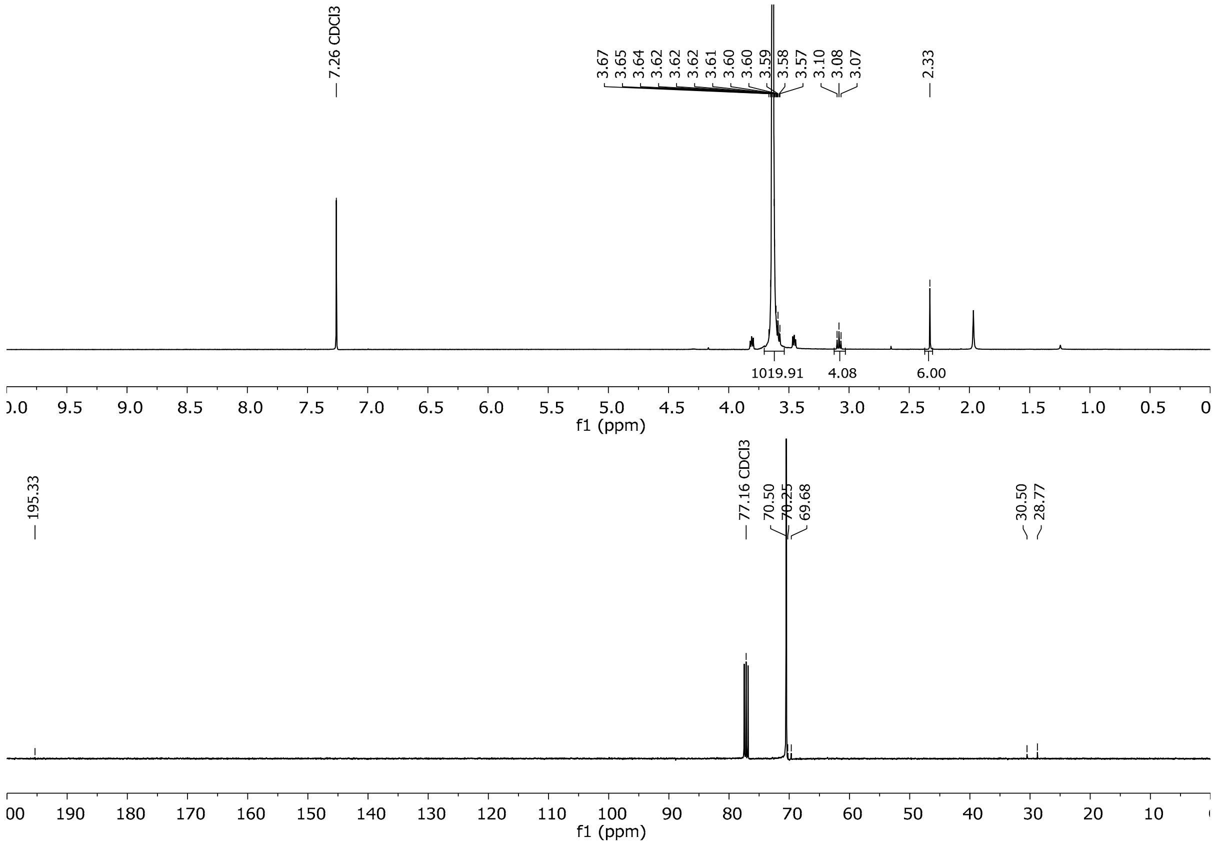

Figure S14. ^{1}H and ^{13}C NMR spectra of PEG10k-SAc in deuterated chloroform.

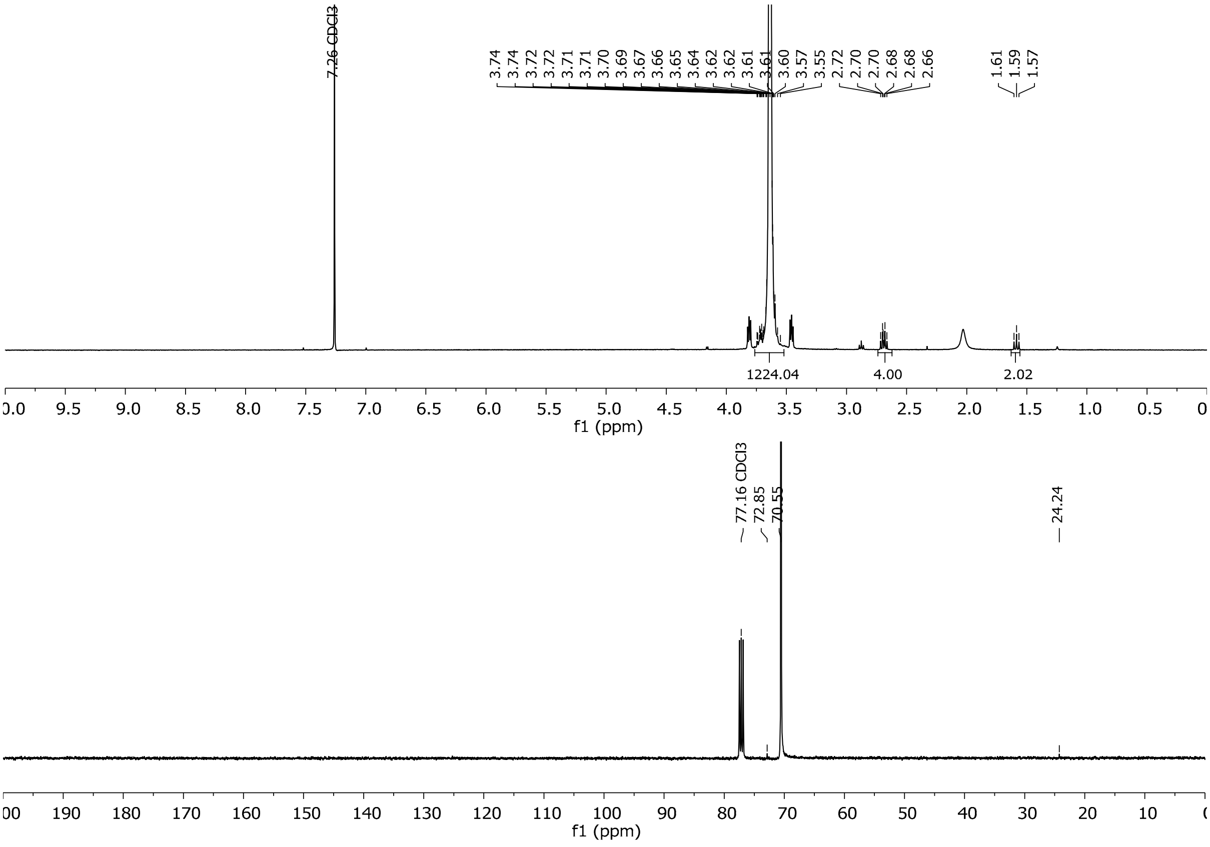

Figure S15. ^{1}H and ^{13}C NMR spectra of PEG10k-SH in deuterated chloroform.

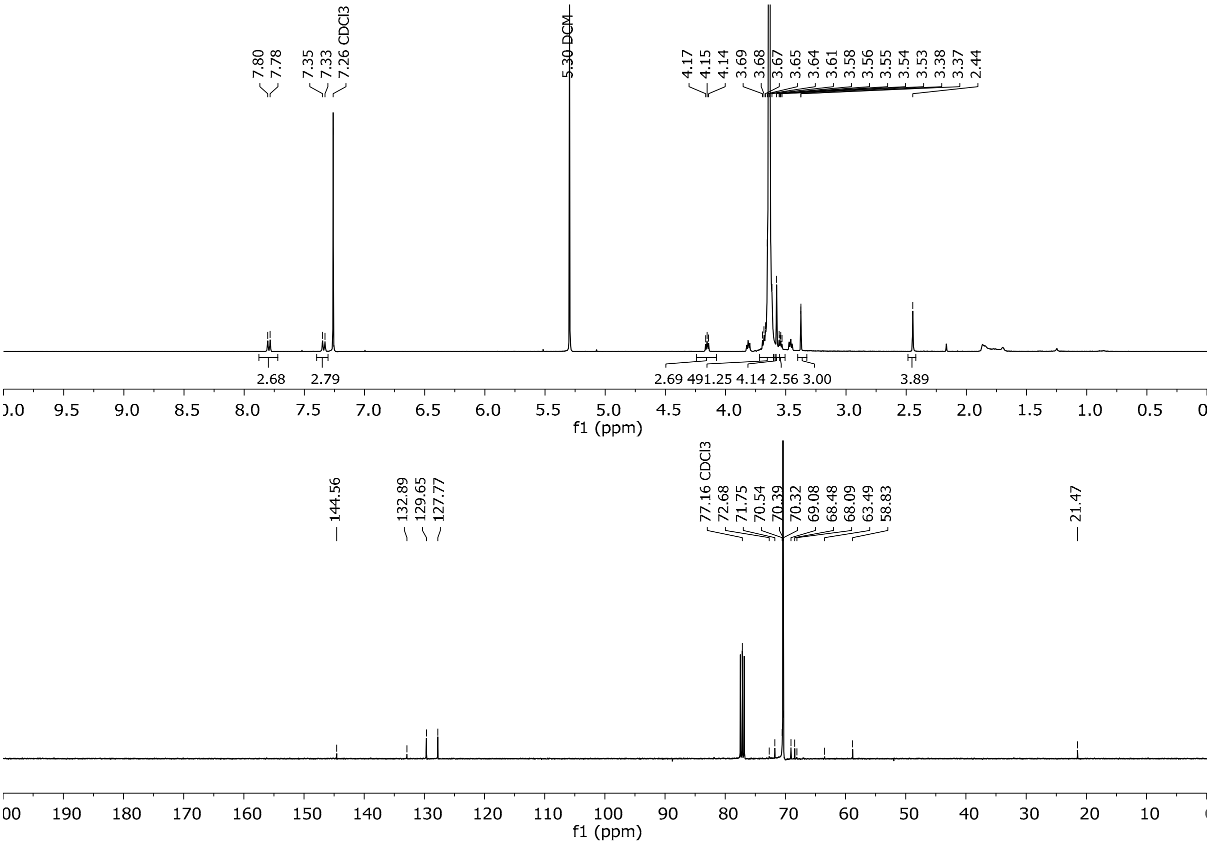

Figure S16. ^{1}H and ^{13}C NMR spectra of mPEG5k-OTs in deuterated chloroform.

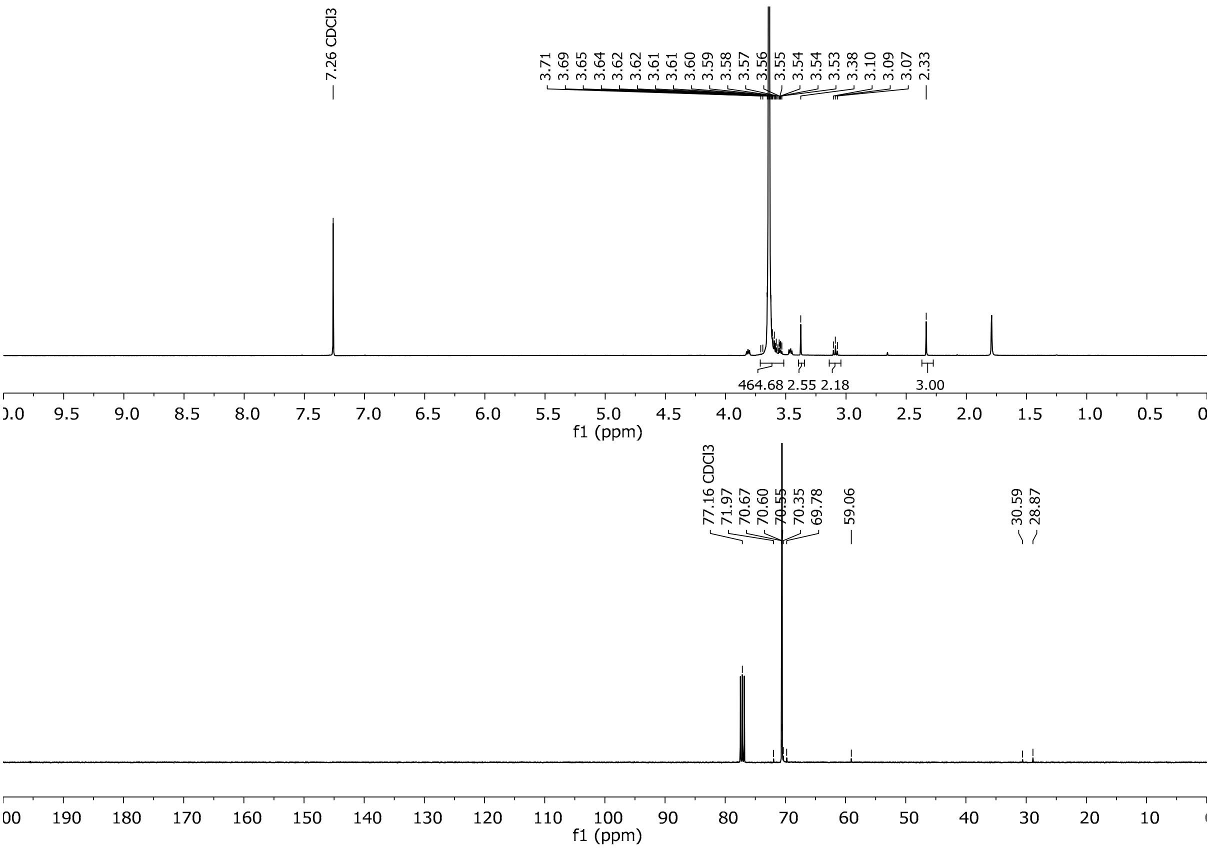

Figure S17. ^{1}H and ^{13}C NMR spectra of mPEG5k-SAc in deuterated chloroform.

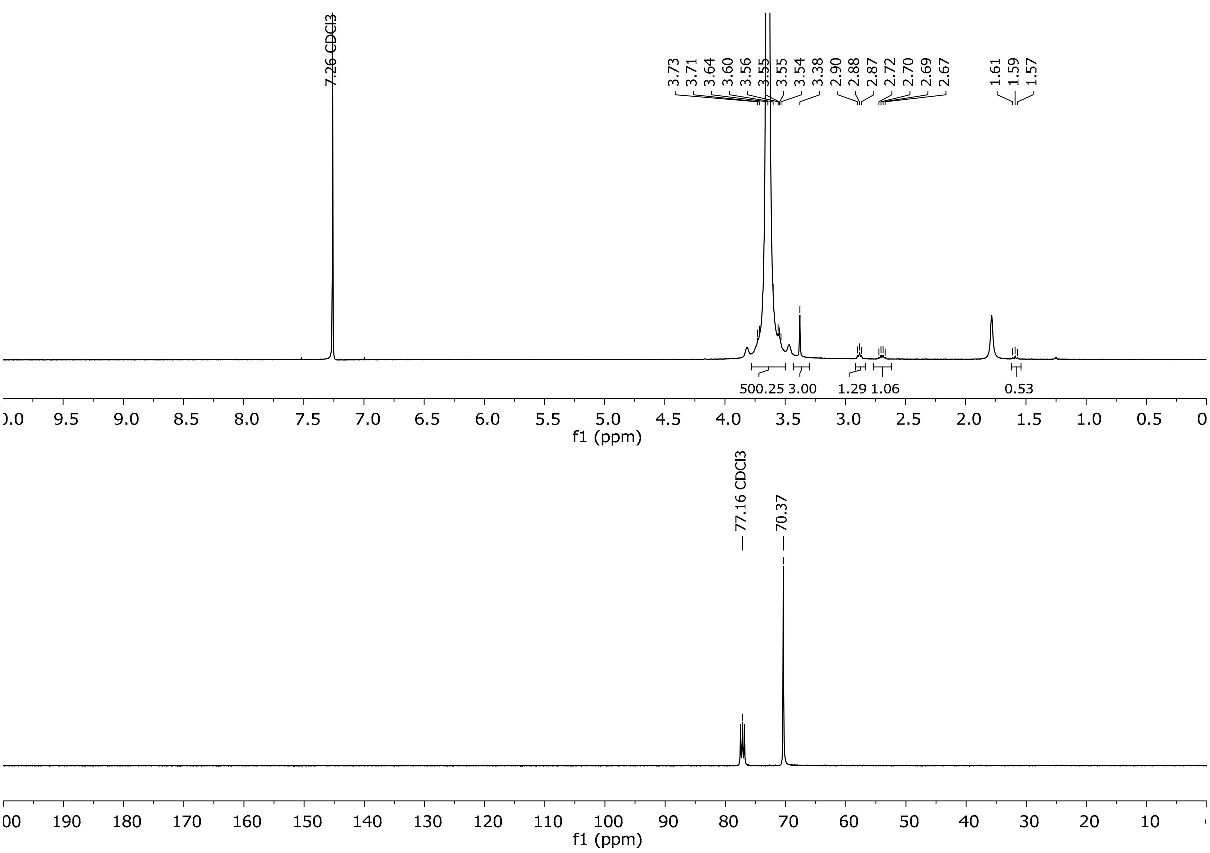

Figure S18. ^{1}H and ^{13}C NMR spectra of mPEG5k-SH in deuterated chloroform.

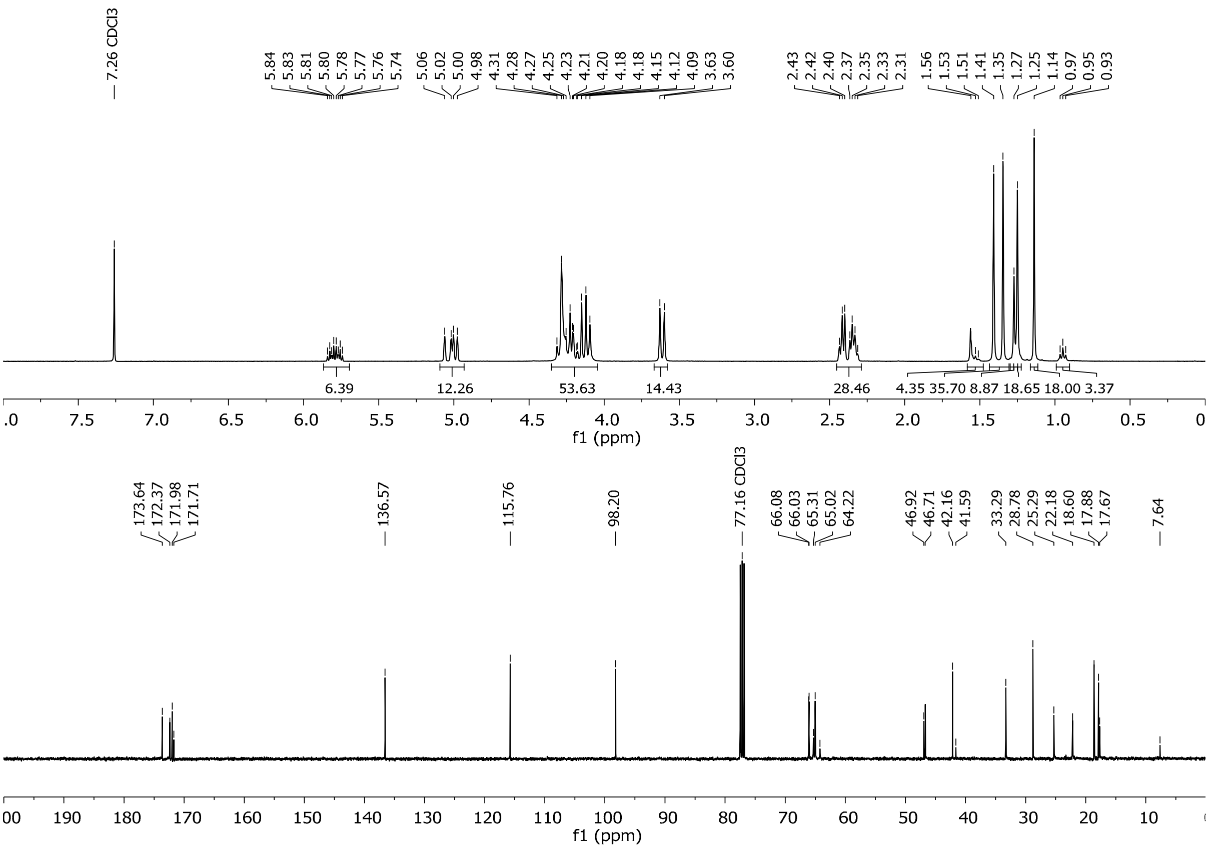

Figure S19. ^{1}H and ^{13}C NMR spectra of TMP-G2-(Acetonide)$_6$(ene)$_6$ in deuterated chloroform.

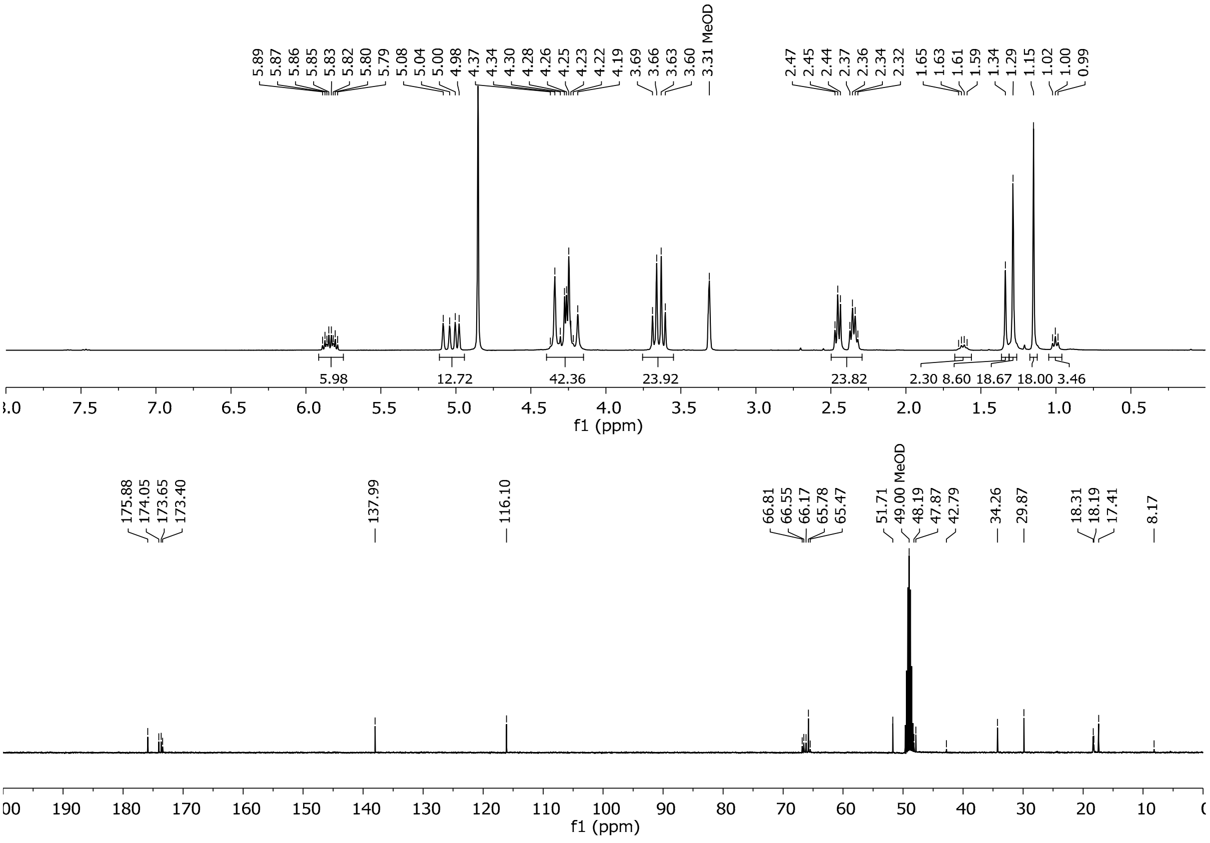

Figure S20. ^{1}H and ^{13}C NMR spectra of TMP-G2-(OH)$_{12}$(ene)$_6$ in deuterated methanol.

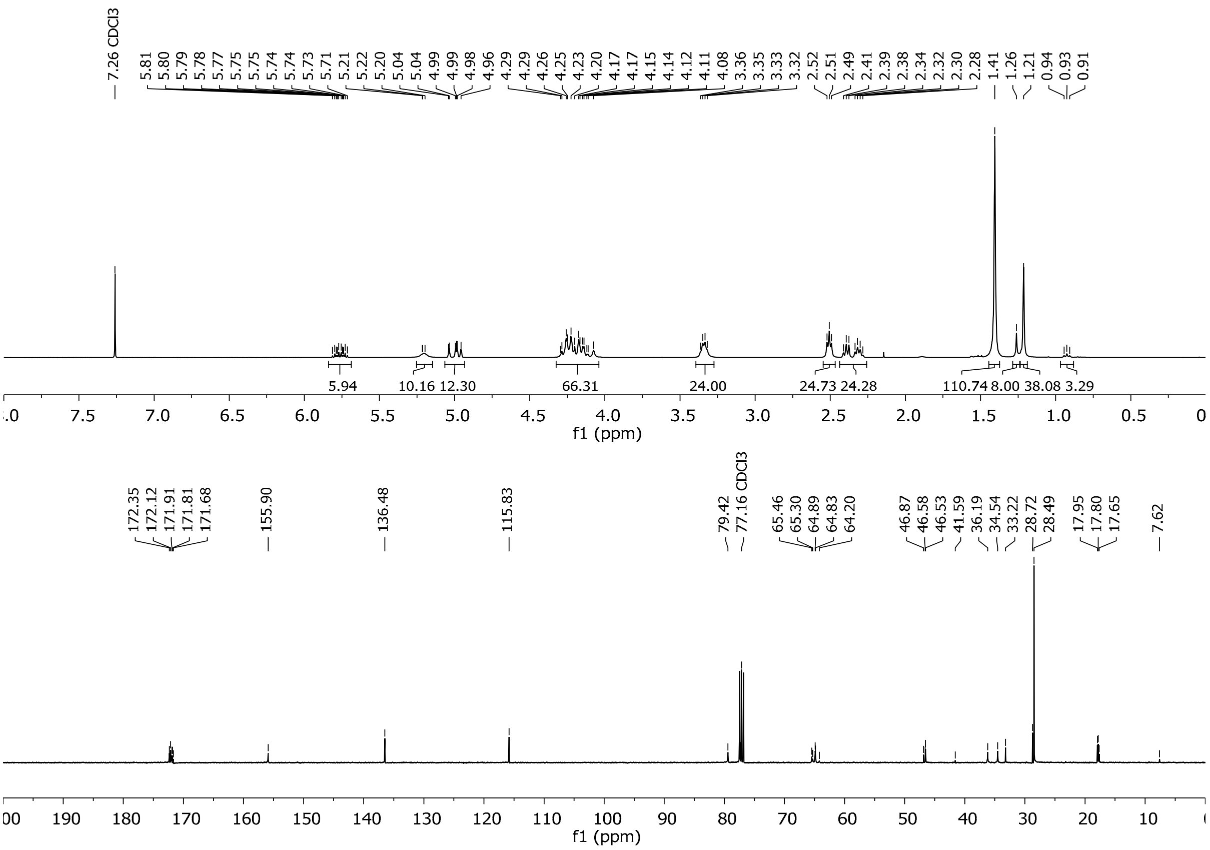

Figure S21. ^{1}H and ^{13}C NMR spectra of TMP-G2-$(NHBoc)_{12}(ene)_6$ in deuterated chloroform.

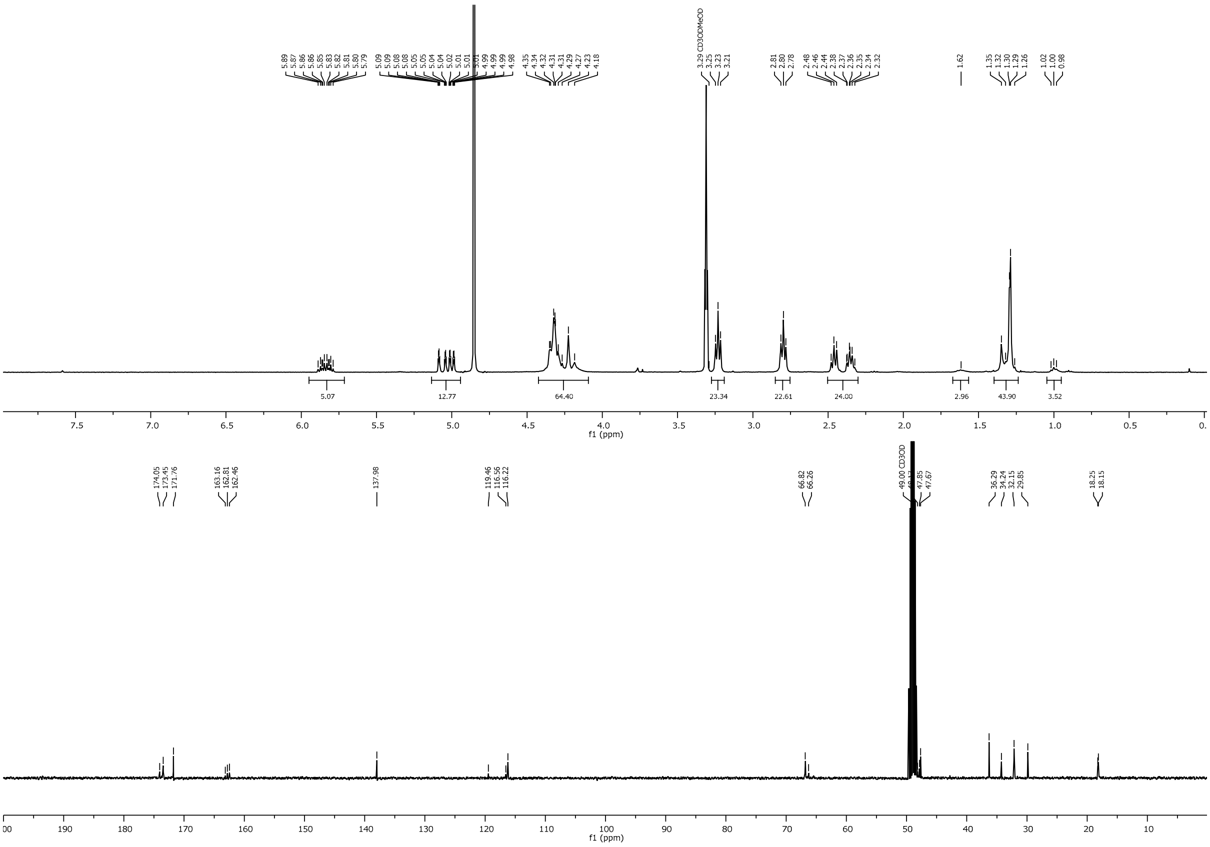

Figure S22. ^{1}H and ^{13}C NMR spectra of TMP-G2-$(NH_3^{+}TFA^{-})_{12}(ene)_6$ in deuterated methanol.

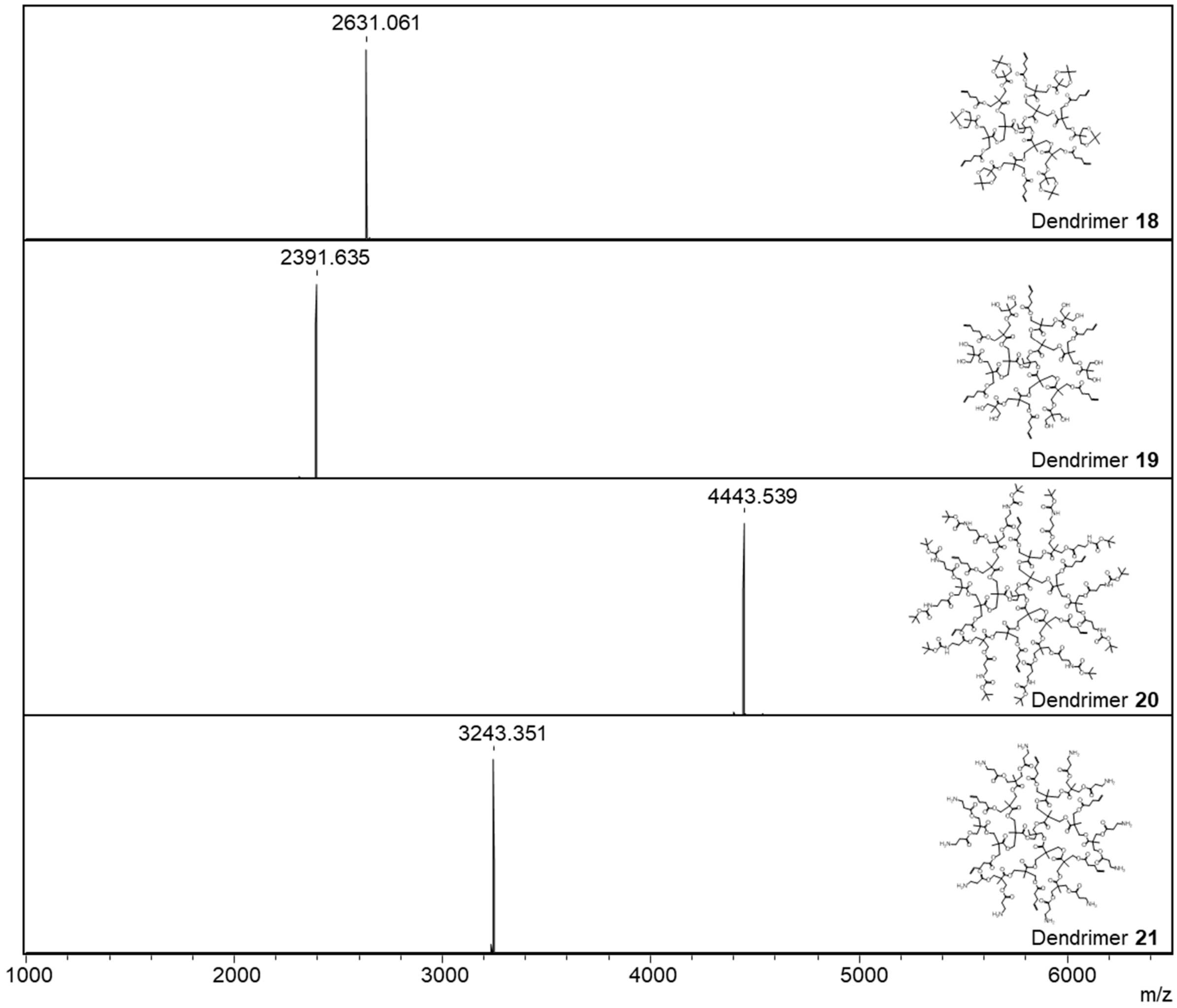

Figure S23. MALDI-ToF-MS of dendrimers **18**, **19**, **20** and **21** in DCTB, DHB, DCTB and DHB respectively.

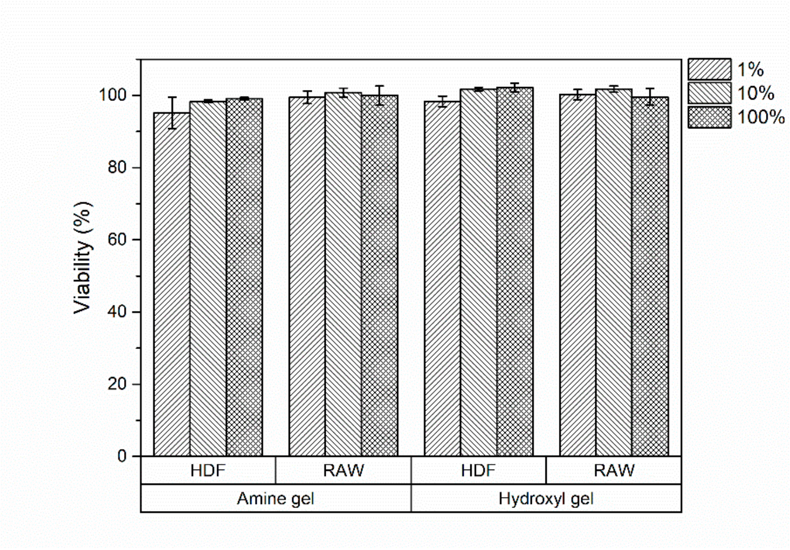

Figure S24. Cytotoxicity evaluation of hydrogel leach out and dilution of the leach out towards HDF and RAW determined by AlamarBlue assay.

References

1. Garcia-Gallego S, et al. Fluoride-promoted esterification with imidazolide-activated compounds: a modular and sustainable approach to dendrimers. *Angew. Chem. Int. Ed. Engl.* **2015**; *54 (8)*, 2416-2419.
2. Salmon-Chemin L, et al. 2- and 3-Substituted 1,4-Naphthoquinone Derivatives as Subversive Substrates of Trypanothione Reductase and Lipoamide Dehydrogenase from Trypanosomacruzi: Synthesis and Correlation between Redox Cycling Activities and in Vitro Cytotoxicity. *J. Med. Chem.* **2001**; *44 (4)*, 548-565.
3. Stenström P, et al. Synthesis and in Vitro Evaluation of Monodisperse Amino-Functional Polyester Dendrimers with Rapid Degradability and Antibacterial Properties. *Biomacromolecules*. **2017**; *18 (12)*, 4323-4330.
4. Ihre H, et al. Double-Stage Convergent Approach for the Synthesis of Functionalized Dendritic Aliphatic Polyesters Based on 2,2-Bis(hydroxymethyl)propionic Acid. *Macromolecules*. **1998**; *31 (13)*, 4061-4068.
5. Garcia-Gallego S, Stenström P, Mesa-Antunez P, Zhang Y, Malkoch M. Synthesis of heterofunctional polyester dendrimers with internal and external functionalities as versatile mulitpurpose platforms. *Biomacromolecules.* **2020**; *21*, 4273-4279
